# An update of the molecular mechanisms underlying anthracycline induced cardiotoxicity

**DOI:** 10.3389/fphar.2024.1406247

**Published:** 2024-06-26

**Authors:** Sicong Xie, Yuwei Sun, Xuan Zhao, Yiqun Xiao, Fei Zhou, Liang Lin, Wei Wang, Bin Lin, Zun Wang, Zixuan Fang, Lei Wang, Yang Zhang

**Affiliations:** ^1^ Department of Rehabilitation Medicine, School of Acupuncture-Moxibustion and Tuina and School of Health Preservation and Rehabilitation, Nanjing University of Chinese Medicine, Nanjing, China; ^2^ Department of General Surgery, Jiangsu Province Hospital of Chinese Medicine, Affiliated Hospital of Nanjing University of Chinese Medicine, Nanjing, China; ^3^ College of Electronic and Optical Engineering and College of Flexible Electronics, Future Technology, Nanjing University of Posts and Telecommunications, Nanjing, China; ^4^ Key Laboratory of Intelligent Pharmacy and Individualized Therapy of Huzhou, Department of Pharmacy, Changxing People’s Hospital, Huzhou, China

**Keywords:** anthracycline, cardiotoxicity, mitochondria, DNA, signal pathway

## Abstract

Anthracycline drugs mainly include doxorubicin, epirubicin, pirarubicin, and aclamycin, which are widely used to treat a variety of malignant tumors, such as breast cancer, gastrointestinal tumors, lymphoma, etc. With the accumulation of anthracycline drugs in the body, they can induce serious heart damage, limiting their clinical application. The mechanism by which anthracycline drugs cause cardiotoxicity is not yet clear. This review provides an overview of the different types of cardiac damage induced by anthracycline-class drugs and delves into the molecular mechanisms behind these injuries. Cardiac damage primarily involves alterations in myocardial cell function and pathological cell death, encompassing mitochondrial dysfunction, topoisomerase inhibition, disruptions in iron ion metabolism, myofibril degradation, and oxidative stress. Mechanisms of uptake and transport in anthracycline-induced cardiotoxicity are emphasized, as well as the role and breakthroughs of iPSC in cardiotoxicity studies. Selected novel cardioprotective therapies and mechanisms are updated. Mechanisms and protective strategies associated with anthracycline cardiotoxicity in animal experiments are examined, and the definition of drug damage in humans and animal models is discussed. Understanding these molecular mechanisms is of paramount importance in mitigating anthracycline-induced cardiac toxicity and guiding the development of safer approaches in cancer treatment.

## 1 Introduction

In the 1940s, first anthracycline, Daunorubicin (DAU), was discovered by the *Streptomyces* bacteria, marking a significant breakthrough in oncology (Marco et al., 1981). Clinical trials demonstrated remarkable success in using DAU to treat acute leukemia (Tan et al., 1967). Subsequently, the exploration of other anthracycline antibiotics led to the discovery of the anticancer drug doxorubicin (DOX), a precursor of DAU, which not only proved effective for leukemia but also exhibited therapeutic properties against most solid tumors (Arcamone et al., 1969). Currently, widely used anthracycline compounds in clinical practice include DOX, Pirarubicin (THP), Idarubicin (IDU), and Epirubicin (EPI). Anthracyclines remain frontline agents in many cancer chemotherapy regimens.

However, despite their ability to improve long-term survival in cancer patients, anthracycline-type drugs can induce cardiotoxicity after reaching a certain cumulative dose (Sawicki et al., 2021). Middleman observed a dose-dependent relationship between anthracycline drug dosage and cardiac damage in 1971 (Middleman et al., 1971). Clinically, acute anthracycline-induced cardiotoxicity is characterized by electrocardiographic abnormalities (20%–30%), neutropenia (6%), arrhythmias (3%), and in some patients reversible cardiac insufficiency (Middleman et al., 1971). Anthracycline-induced chronic cardiotoxicity may occur months or years after initial therapy and can be categorized as early-onset or late-onset. Early-onset chronic cardiotoxicity usually occurs within 1 year after completion of anthracycline therapy in adults, whereas late-onset chronic cardiotoxicity may occur decades after treatment and is commonly seen in pediatric patients who have received chemotherapy (van Dalen et al., 2011). The incidence of doxorubicin-induced cardiotoxicity, primarily congestive heart failure, is strongly dependent on the cumulative dose. Studies have shown that the incidence of congestive heart failure was 3%, 7%, and 18% at cumulative doses of 400, 550 and 700 mg/m^2^, respectively. A combined analysis of three studies of 630 patients with breast and lung cancer further showed that the incidence of congestive heart failure was even higher, with rates of 4.7%, 26% and 48% at cumulative doses of 400, 550, and 700 mg/m^2^, respectively (Silber, 2004). Due to the limited terminal differentiation of cardiac myocytes, they are vulnerable to the effects of anthracycline drugs, leading to cardiac injury (Khouri et al., 2012).

## 2 Types of cardiac damage induced by anthracycline-type drugs

**FIGURE 1:**
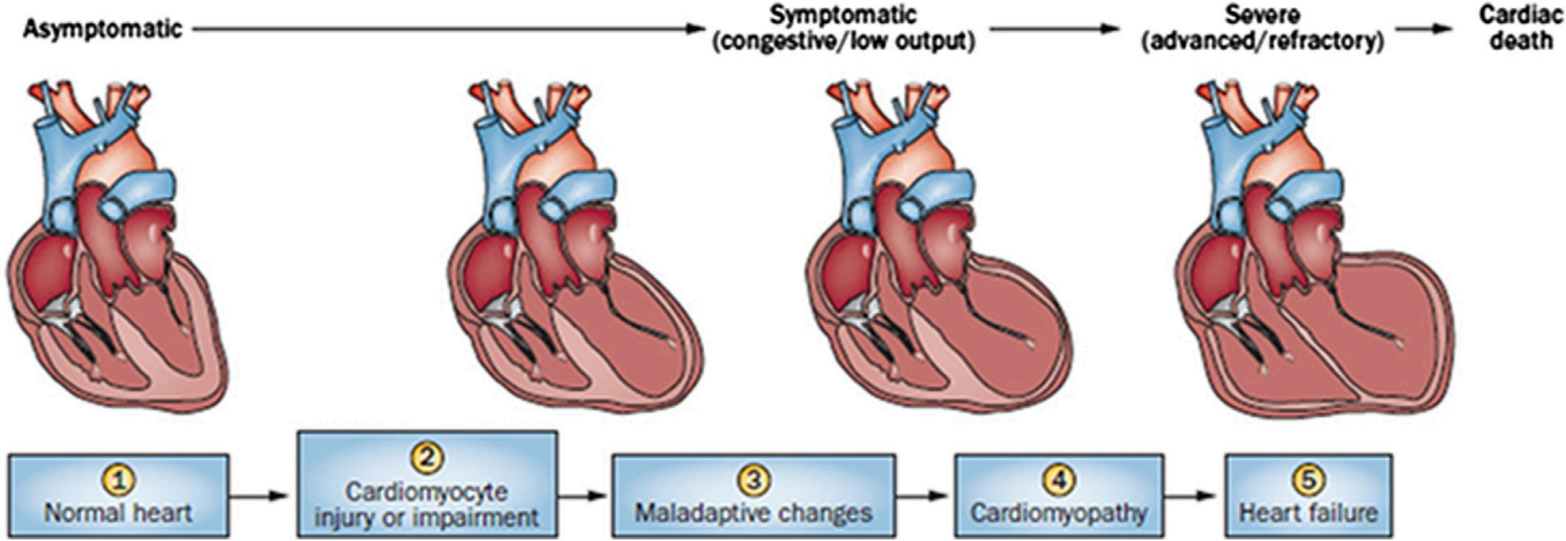
Stages in the course of Anthracycline-induced ventricular dysfunction. (1) Primary prevention is possible at this stage by reducing risk factors in high-risk populations (such as those receiving anticancer therapy). (2) Secondary prevention is possible at this stage to reduce the effects of the treatment-induced injury. (3) Secondary prevention is also possible at this stage. (4) Clinically significant conduction and rhythm abnormalities might be observed. (5) Radical therapies might be required at this stage (such as heart transplant) if there is failure of medical management. Preventive strategies are progressively less effective as the toxicity increases. Treatment strategies have a greater impact when used to treat themore-diseased heart, but have longer effects if initiated early. Reproduced with permission from Madonna (2017), Copyright 2017 Sociedad Española de Cardiología. Published by Elsevier España S.L.U. All rights reserved.

Cardiac damage caused by anthracycline-type drugs is the result of various molecular processes, primarily centered on alterations in cardiac myocyte function and pathological cell death (Nebigil and Désaubry, 2018). The initial manifestation of cardiac damage is subclinical myocardial injury, progressing to early asymptomatic reductions in left ventricular ejection (LVEF) fraction and ultimately leading to severe heart failure (Yeh and Bickford, 2009) (Figure 1).

Anthracycline-induced cardiac damage can be categorized as acute and chronic, depending on the initial symptom presentation (Cardinale et al., 2015). Acute cardiac injury typically manifests within the first few days of administering anthracycline drugs, presenting as acute myocarditis with myocardial cell injury, inflammatory infiltrates, and interstitial edema (Ferrans, 1978). Clinically, acute cardiac damage manifests as changes in electrocardiograms, neutropenia, arrhythmias, and, in some patients, reversible cardiac dysfunction (Shan et al., 1996). Chronic cardiac damage can develop months or years after initial treatment and can be classified as either early or late onset. Early-onset chronic cardiac damage usually arises within the first year following anthracycline drug treatment, whereas late-onset chronic cardiac damage can manifest decades later, particularly in pediatric patients. (Cardinale et al., 2015). Chronic cardiac damage is characterized by cardiomyopathy or congestive heart failure (CHF), along with cardiac enlargement, reduced LVEF fraction, ST-T segment changes, and cellular-level features such as cytoplasmic and mitochondrial swelling, organelle rupture, cardiomyocyte death, and myofibrillar disarray leading to cytoplasmic vacuolization (Berry and Jorden, 2005). When the cumulative dose of anthracycline drugs reaches 550 mg/m^2^, approximately 26% of patients experience irreversible LVEF reduction, and more than 7% develop heart failure (Swain et al., 2003).

## 3 Molecular mechanisms of anthracycline-induced cardiac damage

Currently, the primary mechanism of anthracycline-induced cardiotoxicity is believed to involve the inhibition of Topoisomerase (TOP) 2β (Henriksen, 2018). Other mechanisms include disruptions in iron ion metabolism, mitochondrial dysfunction, myocardial fiber dissolution, sarcoplasmic reticulum calcium imbalance, oxidative stress, and cell apoptosis (Bansal et al., 2019). While many mechanisms have been associated with cardiac toxicity, the exact mechanisms remain unclear.

### 3.1 TOP inhibition

**FIGURE 2:**
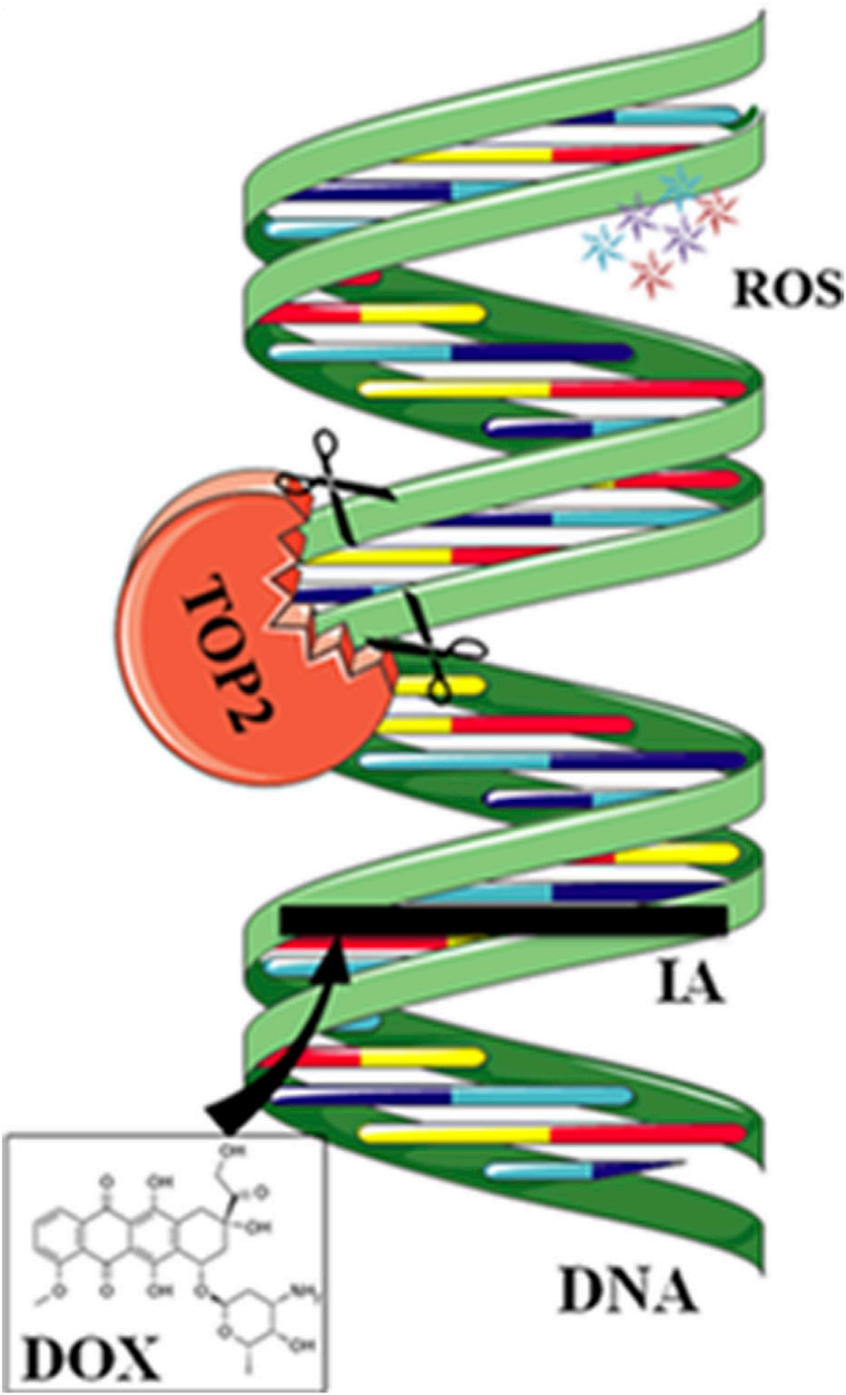
Mechanisms of action of doxorubicin (DOX). DOX intercalates between strands of DNA double helix. The formation of a ternary complex (TOP2-DOX-DNA) prevents enzyme turnover. The latter blocks the catalytic cycle after DNA is cleaved and before DNA relegation. DOX biotransformation results in the formation of ROS. Reproduced with permission from Corremans et al. (2019), Copyright 2018 John Wiley and Sons Australia, Ltd.

Anthracycline drugs act by binding to DNA TOPs, inserting into DNA, promoting DNA strand breaks, and causing cardiac damage (Tewey et al., 1984). TOPs are enzymes that regulate DNA replication, transcription, recombination, and chromatin remodeling by inducing temporary single or double-strand breaks when they open DNA strands. In human cells, there are two Top2 isoforms, Top2α and Top 2β. Top2α is expressed at high levels during the cell cycle in undifferentiated and proliferating cells, including those of tumors, with varying levels. In contrast, Top2β is expressed in quiescent and differentiated cells, such as those found in cardiac muscle, maintaining consistent levels throughout the cell cycle (Wang, 2002) (Figure 2).

DOX binds to Top2α, arresting tumor cells in the G_1_/G_2_ phase and inhibiting DNA replication, leading to apoptosis. However, anthracycline drugs also bind to Top2β, inhibiting DNA replication, resulting in apoptosis of cardiac muscle cells. Experiments have shown that specific knockout of the Top2β gene in mouse hearts can alleviate DOX-induced heart failure. Compared to the control group, double-strand DNA breaks decrease, suppressing apoptosis (Zhang et al., 2012). DZR besides its iron-chelating function, can inhibit Top2β activity, which may contribute to its cardiac protection (Vavrova et al., 2013). Crystal structure analysis shows that DZR stabilizes ATP in the ATPase domain of Top2β, preventing the reopening of the Top2β subunit and locking the Top2β monomer in a closed dimeric structure. This, in turn, prevents anthracycline drugs from binding to the TOP-DNA complex and inducing DNA damage. (Classen et al., 2003; Lee et al., 2017). Researchers have developed anthracycline compounds that specifically target Top2α. While this approach does not affect the expression of Top2β in differentiated cardiac muscle cells, it may potentially impact undifferentiated cardiac progenitor cells expressing Top2α.

In addition to expressing Top2β, cardiac muscle cells also express mitochondrial TOP 1 (Top1mt), which regulates the stability of mtDNA. Mice that lack Top1mt demonstrate mitochondrial defects, such as compromised synthesis of respiratory proteins and disrupted mitochondrial ultrastructure. They are more susceptible to DOX-induced damage compared to normal mice, leading to cardiac injury (Khiati et al., 2014). Therefore, Top1mt has a cardioprotective role, and genetic variations in Top1mt may increase sensitivity to anthracycline cardiotoxicity.

### 3.2 Iron ion metabolism disturbance

Under normal physiological conditions, there is only a minimal amount of biologically active free iron in cardiac muscle cells. Most iron ions are bound to iron proteins to prevent their escape and avoid tissue and cell damage. When there is an excess of iron ions in the cells, anthracycline drugs increase intracellular reactive oxygen species (ROS) and induce oxidative stress reactions, causing cardiac muscle damage (Xu et al., 2005).

When a significant amount of anthracycline drugs accumulates within cells, the anthracycline drug’s core structure, anthraquinone, can be reduced to semiquinone radicals by reduced nicotinamide adenine dinucleotide phosphate dehydrogenase (NADPH). Nucleotide phosphate dehydrogenase (NADPH) to semiquinone radicals. These radicals undergo auto-oxidation under aerobic conditions, generating anthraquinone radicals and superoxide anions (O^2-^), leading to intracellular O^2-^ accumulation. Meanwhile, O^2-^ is converted to hydrogen peroxide (H_2_O_2_) by superoxide dismutase (SOD). H_2_O_2_ can react with iron ions through the Fenton reaction, converting H_2_O_2_ into toxic hydroxyl radicals (-OH), thus causing sustained increases in intracellular ROS levels and oxidative stress reactions, leading to cardiac muscle damage (Doroshow and Davies, 1986; Winterbourn, 1995).

**FIGURE 3:**
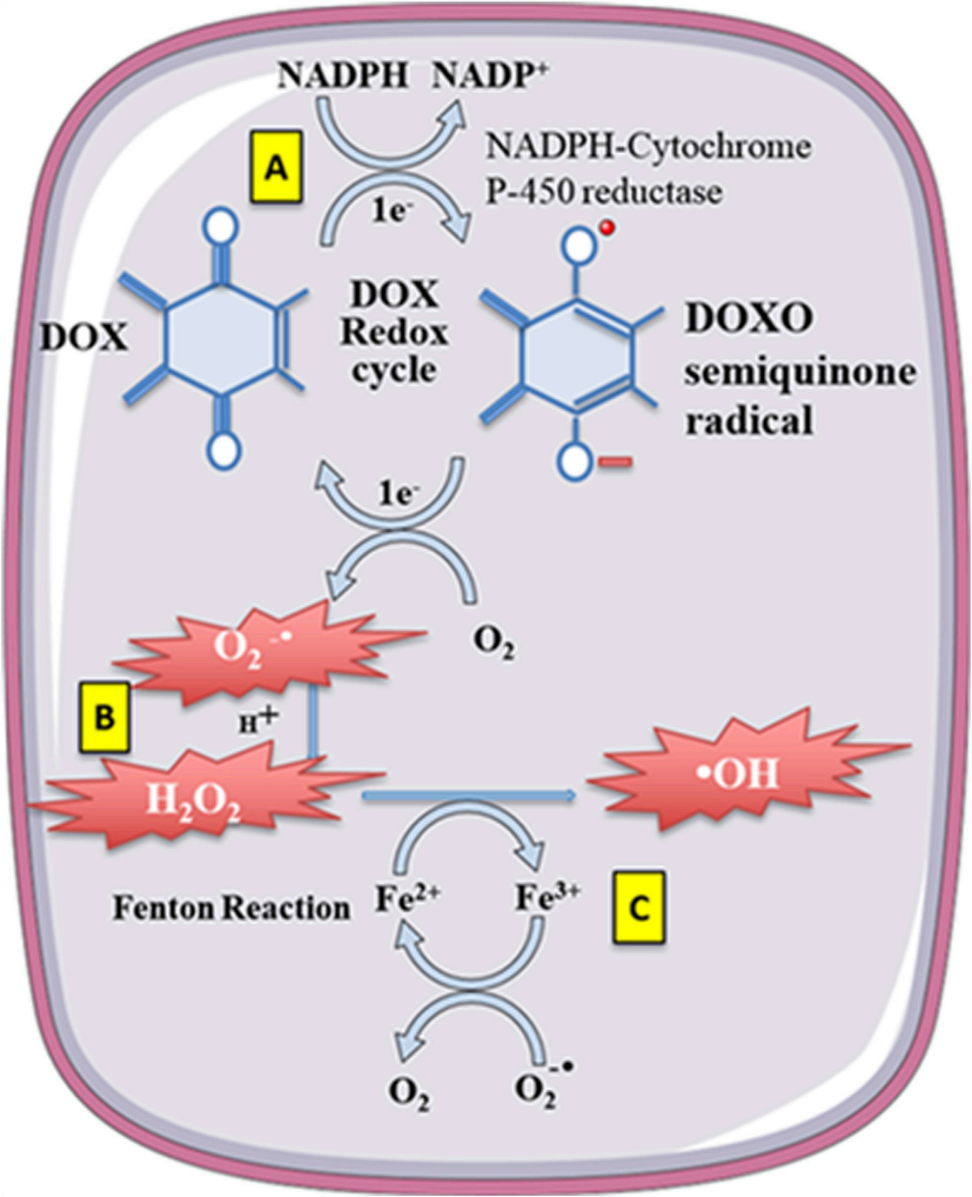
Doxorubicin (DOX) undergoes redoxcycling catalysed by NADPH-Cytochrome P-450 reductase. **(A)** The One-electron (1e^-^) reduction of the quinone compound leads to redox cycling and generation of superoxide anion radical. **(B)** O_2_-undergoes dismutation to form H_2_O_2_ either spontaneously or catalysed by superoxide dismutase. **(C)** H_2_O_2_ then reacts with the transition metal ion Fe2^+^, giving rise to -OH. Free Fe2^+^ istoxic to cells as catalyst in the formation of free radicals from ROS via the Fenton reaction. Reproduced with permission from Corremans et al. (2019), Copyright 2018 John Wiley and Sons Australia, Ltd.

Anthracycline drugs can also directly interact with iron ions, forming complexes in a cyclic manner between Fe^2+^ and Fe^3+^, leading to oxidative stress and mitochondrial dysfunction (Myers et al., 1982). DOX derivatives can also chelate iron ions from cytoplasmic iron-regulatory protein 1 (IRP1) iron-sulfur (Fe-S) clusters, rendering IRP1 inactive. Inactive IRP1 increases the synthesis of transferrin receptor (TF), reducing the synthesis of intracellular iron storage protein (ferritin), disrupting cellular iron homeostasis, ultimately resulting in continued increases in intracellular free iron (Minotti et al., 1998) (Figure 3).

Mitochondrial iron export protein (ATP Binding Cassette Transporter B8, ABCB8) is one of the essential factors affecting mitochondrial iron metabolism during DOX treatment. Studies have reported that DOX can downregulate the expression of ABCB8, leading to increased iron accumulation within mitochondria. Transgenic mice overexpressing ABCB8 exhibit protective effects against DOX-induced cardiac muscle damage. ABCB8 overexpression reduces the iron content in mitochondria, thereby reducing DOX-induced cardiac toxicity (Menon and Kim, 2022). This suggests that reducing intracellular mitochondrial iron ion content may be an effective strategy to prevent anthracycline-induced cardiac toxicity.

Although both *in vitro* and *in vivo* studies confirm an increase in free iron content within cardiac muscle cells after anthracycline treatment, the role of iron disturbance in inducing cardiac damage is still debated. Not all iron chelators have shown efficacy in mitigating anthracycline-induced cardiac damage (Stěrba et al., 2013). DZR, an iron chelator, can reduce anthracycline-induced cardiac damage, but other potent iron chelators, such as deferoxamine, deferiprone, and deferasirox, have yet to show this protective effect (Sultan et al., 2017).

### 3.3 Mitochondrial dysfunction

**FIGURE 4:**
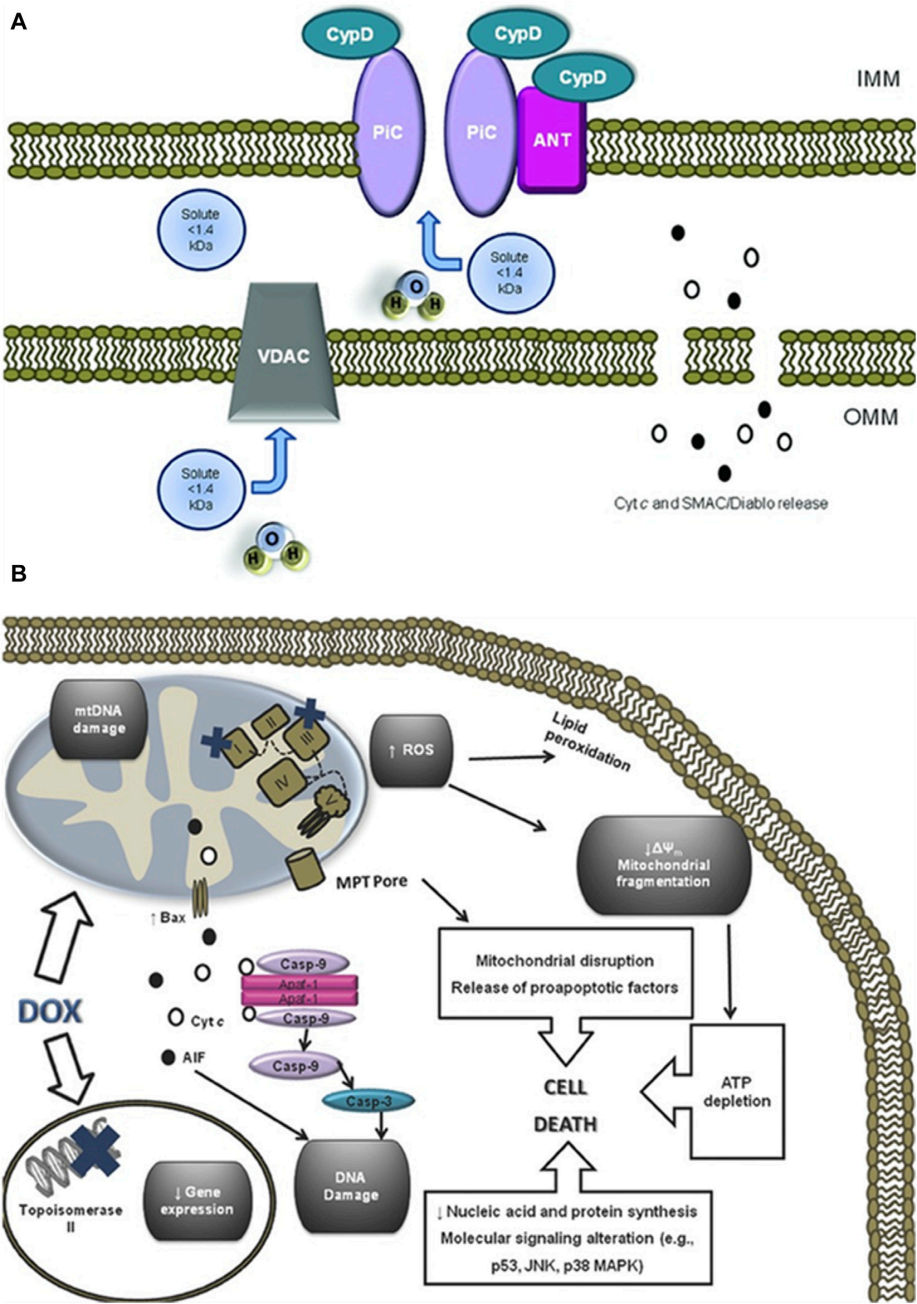
Anthracycline drugs induced changes in mitochondrial membrane permeability and induced cell death. **(A)** Mitochondrial permeability transition pore (MPTP) structure. It was recently proposed that the MPTP is generally considered to be a complex channel composed of several proteins, including voltage-dependent anion channel 1 (VDAC1) in the outer membrane, adenine nucleotide translocase 1 (ANT1) in the inner membrane, and cyclophilin D (CYPD) in the mitochondrial matrix. In addition, the MPTP can be regulated by other components, such as hexokinase (HK), creatine kinase (CK), and peripheral-type benzodiazepine receptors (PBR). Both antiapoptotic and proapoptotic members of the Bcl-2 family modulate the activity of MPTP (antiapoptotic members of the Bcl-2 family, including Bcl-2 and Bcl-XL, inhibit pore openingwhile proapoptotic Bcl-2 family members, such as Bax, Bak, and Bid, can induce MPT pore opening). Also, MPT pore opening can be inhibited by CypD ligands, such as cyclosporin A (CsA). The opening of MPTP leads to a collapse of transmembrane mitochondrial transmembrane potential and favors the release of apoptogenic proteins, such as cytochrome c (Cyt c). **(B)** A large component of DOX induced cardiotoxicity is mediated by a redox cycle on mitochondrial complex I. Increased ROS generation by DOX redox cycle has several negative consequences, such as mitochondrial transmembrane potential disruption, MPTP formation, ATP depletion, and peroxidation of cellular membranes. Marked mitochondrial morphological disturbances induced by DOX include cristae disruption, matrix disorganization, and mitochondrial fragmentation. MPTP -induced outer membrane rupture due to osmotic swelling or permeabilization of the mitochondrial outer membrane mediated by proapoptotic proteins including BAX can lead to the release of cyt c and AIF. DOX also interferes with topoisomerase II, inhibiting DNA replication and preventing the repair of damage DNA strands. Finally, persistent downregulation of gene expression can be another consequence of DOX toxicity. All of these events may lead to cell death. Reproduced with permission from Carvalho et al. (2014), Copyright 2013 Wiley Periodicals, Inc.

As the primary site for cellular energy production and aerobic respiration, mitochondria are primarily composed of the mitochondrial outer membrane, intermembrane space, mitochondrial inner membrane, and mitochondrial matrix (Varga et al., 2015). In addition to their roles in energy conversion, the tricarboxylic acid cycle, oxidative phosphorylation, and calcium storage (Nunes-Nesi et al., 2013), mitochondria also play significant roles in processes such as cell proliferation, metabolism, and apoptosis (Su et al., 2023). An increasing body of research suggests that anthracycline drugs induce cardiac damage associated with mitochondrial dysfunction. When anthracycline drugs enter cardiac muscle cells, they alter mitochondrial membrane permeability, causing the release of cytochrome c (Cyt c) into the cytoplasm, subsequently triggering apoptosis. Additionally, anthracycline drugs can inhibit the activity of mitochondrial inner transcription enzymes, leading to ongoing oxidative stress in damaged mitochondria and exacerbating apoptosis (Parker et al., 2001) (Figure 4).

#### 3.3.1 Mitochondrial membrane permeability

Cardiolipin is an essential component of the mitochondrial inner membrane. Anthracycline drugs exhibit a strong affinity for cardiolipin, forming irreversible complexes through positive and negative charge interactions and accumulating on the mitochondrial inner membrane (Parker et al., 2001). When the complex accumulates to levels exceeding 50–100 uM in the mitochondria, it leads to a significant increase in mitochondrial reactive ROS (Sarvazyan, 1996). High levels of ROS can result in oxidative reactions in mitochondrial proteins and lipids, leading to mitochondrial dysfunction and changes in mitochondrial membrane potential. Studies have shown that a decrease in mitochondrial membrane potential is present in almost all apoptotic cells and that this alteration occurs before the morphology of apoptotic cells changes, and the decrease in membrane potential is mainly due to the mitochondrial membrane permeability The decrease in membrane potential is mainly due to the permeability of the mitochondrial membrane (Zaib et al., 2022). Mitochondrial membrane permeability is regulated by the mitochondrial permeability transition pore (MPTP), which is located between the inner and outer mitochondrial membranes. It is generally considered to be a complex channel composed of several proteins, including voltage-dependent anion channel 1 (VDAC1) in the outer membrane, adenine nucleotide translocase 1 (ANT1) in the inner membrane, and cyclophilin D (CYPD) in the mitochondrial matrix.

VDAC1 is primarily located in the outer mitochondrial membrane of eukaryotic organisms, with a molecular weight of 35 KDa. In its open state, it can mediate the passage of ions and small molecules such as nucleotides (Benz et al., 1992). VDAC1 can also mediate the entry of respiratory chain substrates such as pyruvate, succinate, and malate into the mitochondria (Shoshan-Barmatz et al., 2010). VDAC1 is closely associated with the development of many diseases. Research has shown that VDAC1 is highly expressed in cancer cells (Shoshan-Barmatz et al., 2015). Arif et al. silenced VDAC1 expression by means of interfering RNA (siRNA) in cancer cells and found that siVDAC1 reduced VDAC1 levels by approximately 90% in lung cancer cells A549 and H358, prostate cancer cells PC-3, colon cancer cells HCT116, glioblastoma U87, hepatocellular carcinoma cells HepG2, and pancreatic cancer cells Panc-1 when the siVDAC1 was only at nanomolar concentrations. After transfection for 144 h, VDAC1 silencing persisted and significantly inhibited the growth of cancer cells. The mechanism of action is that when VDAC1 is downregulated in tumor cells, it leads to a decrease in mitochondrial membrane potential and adenosine triphosphate (ATP) levels, disrupting the exchange of substances between mitochondria and the cytoplasm, thereby inducing apoptosis. Furthermore, in a mouse model of lung cancer, intraperitoneal injection of si-VDAC1 not only inhibited tumor growth but also resulted in tumor disappearance. Therefore, this study suggests that inhibiting the overexpression of VDAC1 could be a novel therapeutic target for cancer treatment (Arif et al., 2017). Meanwhile, studies have reported that VDAC1 is also involved in the development of diabetic nephropathy. Gong et al. replicated a rat model of diabetic nephropathy using streptozotocin injections, and the results showed that VDAC1 expression significantly increased in the renal tissues of the model group, along with the occurrence of oxidative stress and severe interstitial nephritis (Gong et al., 2009). In an *in vitro* experiment of diabetic cardiomyopathy (DCM), it was found that when cardiomyocytes were transfected with lncRNA H19, the expression of miR-675 inside the cells decreased. Luciferase reporter gene experiments showed that miR-675 intertargets with VDAC1. When miR-675 inhibitor was transfected, VDAC1 expression increased, promoting cell apoptosis. When lncRNA H19 was highly expressed, it reduced the expression of VDAC1 inside the cells, inhibiting the occurrence of cardiomyocyte apoptosis. This experimental result suggests that the lncRNA H19/miR-675 axis regulates apoptosis by targeting VDAC1 (Li X. et al., 2016). Xu et al. found that in an experiment inducing apoptosis of mouse foot cell by DOX, the IP3R/glucose-regulated protein 75 (Grp75)/VDAC1/mitochondrial calcium uniporter (MCU) signaling pathway was activated, resulting in Ca^2+^ overload in mitochondria and an increase in cleaved caspase-3 levels, leading to apoptosis (Xu et al., 2018). No relevant research on the association between anthracycline drug-induced heart damage and VDAC1 was found in the literature.

ANT1 is located in the mitochondrial inner membrane, with a molecular weight of 33 kDa, and it accounts for 10% of the total mitochondrial proteins (Clémençon et al., 2013; Palmieri, 2013; Di Noia and Cirigliano, 2014). ANT1 is the sole ATP/adenosine diphosphate (ADP) transporter in eukaryotic cells, translocates back and forth between the inner mitochondrial membrane and the mitochondrial matrix to perform a translocation function, with its substrates being deoxyadenosine diphosphate (dADP), ADP, and ATP (Mielke et al., 2014). A study have shown that the protective role of Cyclosporine-A (CsA) on lung I/R injury depends on the inhibition of MPTP and CytC release, suppression of the activation of mitochondrial apoptosis pathway and the expressions of apoptotic-related proteins, as well as the decreased expression levels of ANT1 and VDAC1 (Li et al., 2017). Many inflammatory diseases are associated with abnormal activation of the NLRP3 inflammasome. The Src homology two domain of protein tyrosine phosphatase 2 (SHP2) in the cytoplasm can be transferred to mitochondria when an inflammatory stimulus enters the cell, interacts with ANT1, and leads to its overexpression, resulting in excessive activation of the NLRP3 inflammasome and triggering an inflammatory response (Guo et al., 2017). In a study of inducing myocardial infarction in wild-type (WT) and heart-specific ANT1 transgenic (ANT1-TG) rats, it was found that overexpressing ANT1 significantly reduced the myocardial infarction area and increased the survival rate of infarcted rats. At the same time, it reduced the release of mitochondrial Cyt c and activation of caspase-3. These results suggest that overexpression of ANT1 can compensate for damaged ANT activity, inhibiting apoptosis (Klumpe et al., 2016). Previous research has confirmed the involvement of ANT1 in anthracycline drug-induced heart damage. Matthias et al. found that in mice with DOX-induced cardiac damage, the expression of ANT1 mRNA in cardiac tissues significantly increased, leading to energy transport blockage within mitochondria and causing apoptosis (Schwebe et al., 2015).

CYPD is located in the mitochondrial matrix, with a molecular weight of 20 KDa. As a member of the immunoaffinity family, CYPD plays a crucial role in protein folding in mitochondria and participates in mitochondrial immune suppression. Primary isolation of embryonic fibroblast MEF cells from CYPD knockout mice and subsequent stimulation with H_2_O_2_ revealed that MEF cells lacking CYPD were less sensitive to H_2_O_2_-induced apoptosis compared to WT MEF cells, suggesting that CYPD is involved in oxidative stress-induced cell death (Delbridge et al., 2011). Moreover, when CYPD-deficient mice were subjected to acute middle cerebral artery occlusion and reperfusion, it was found that the infarction area was significantly reduced compared to normal mice. At the same time, the levels of SOD and catalase (CAT) significantly increased, demonstrating the important role of CYPD in ischemic brain injury models (Schinzel et al., 2005). In an experiment inducing acute kidney injury by cisplatin, it was found that when proximal renal tubule cells were specifically deficient in CYPD, it reduced the occurrence of kidney injury. The mechanism behind this was that cisplatin could enter the cells and cause peroxisome proliferator-activated receptor alpha (PPARα) to bind to CYPD. This, in turn, inhibited PPARα nuclear translocation and transcription, leading to fatty acid oxidation and apoptosis. When CYPD was absent, PPARα nuclear expression and transcription activity were maintained, preventing fatty acid oxidation and intracellular lipid accumulation, thereby exerting a protective effect on cells (Jang et al., 2020). A wealth of research has confirmed the involvement of CYPD in the development of anthracycline drug-induced cardiac damage. Dhingra et al. found that in a model of DOX-induced primary rat cardiac cell damage, the expression of the CYPD gene and protein increased after DOX treatment. This led to a decrease in mitochondrial membrane permeability, calcium accumulation in the mitochondria, and cell apoptosis. Further investigation into the mechanism found that DOX could activate the nuclear transcription factor (NF-κB)/Bcl-2 interacting protein 3 (Bnip3) signaling pathway, leading to an increase in CYPD expression and apoptosis (Dhingra et al., 2020).

A large body of research has confirmed that Bcl-2 family proteins regulate MPTP (Orsi et al., 2017). The Bcl-2 family proteins can be categorized into anti-apoptotic proteins and pro-apoptotic proteins. Anti-apoptotic proteins include Bcl-2, Mcl-1, and Bcl-xL, while pro-apoptotic proteins include Bim, Bid, Bak, and Bax. Most Bcl-2 family proteins have a hydrophobic region at the C-terminus of their peptide chains, allowing them to be positioned on the membrane of organelles. Additionally, the peptide chains of Bcl-2 family proteins contain four conserved helical domains, namely, BH4, BH3, BH2, and BH1, arranged from N-terminus to C-terminus. Among them, anti-apoptotic proteins highly homologous to Bcl-2 contain all four structural domains, making them Bcl-2-like proteins. Furthermore, pro-apoptotic proteins Bax and Bak also contain all four structural domains. Proteins containing only the BH3 domain are referred to as “BH3-only” proteins and are often pro-apoptotic. Their BH3 domain can bind to the hydrophobic groove formed by the BH1, BH2, and BH3 domains of anti-apoptotic proteins, forming heterodimers that control cell survival and apoptosis (Delbridge et al., 2016).

When anthracycline drugs enter cells, they bind to VDAC1 through Bax, leading to the opening of MPTP, a decrease in membrane potential, and the promotion of apoptosis (Kumarswamy and Chandna, 2009). The primary mechanism by which Bax affects VDAC1 is by altering the structure of VDAC1, leading to the loss of voltage dependence and selectivity for ion permeation, resulting in the release of Cyt c from the inner membrane. Bax can also interact with ANT1, forming a channel in the lipid bilayer. When Bcl-2 binds to ANT1, it inhibits the formation of the Bax-ANT1 channel, preventing changes in mitochondrial membrane permeability (Kroemer et al., 2007). Experimental evidence has shown that CYPD binds to Bcl-2 in a specific manner, and overexpression of Bcl-2 can suppress truncated Bid (tBid)-induced Cyt c release. In cells with normal Bcl-2 expression, the addition of a CYPD inhibitor can restore tBid-induced Cyt c release. These results strongly suggest that CYPD can be regulated through Bcl-2 (Eliseev et al., 2009).

Changes in mitochondrial membrane permeability result in the release of Cyt c into the cytoplasm, initiating apoptosis. Cyt c binds to apoptotic protease activating factor-1 (Apaf-1) in the cytoplasm, forming an apoptosome. Subsequently, the apoptosome recruits caspase-9 through the caspase recruitment domain (CARD) and caspase-3 activation, ultimately leading to cell apoptosis (Buttke and Sandstrom, 1994). Therefore, the release of Cyt c serves as a pivotal point for apoptosis initiation and is an essential marker for detecting mitochondrial pathway-induced cell apoptosis.

#### 3.3.2 Mitochondrial transcription enzyme

Additional studies have found that anthracycline drugs can also cause mitochondrial dysfunction by inhibiting the activity of mitochondrial transcription enzymes. These transcription enzymes mainly include Top1mt and mitochondrial transcription factors (Zhang et al., 2012), and mitochondrial transcription factors include peroxisome proliferator-activated receptor-gamma coactivator-1α and 1β (PGC1α and PGC1β), Nuclear respiratory factor-1 (NRF1), mitochondrial transcription factor A (TFAM) (Yin et al., 2018), and Tumor suppressor protein 53 (p53). Research has found that histone deacetylase Sirtuin1 (SIRT1) plays a protective role against anthracycline-induced cardiac toxicity and its mechanism involves SIRT1 activating PGC1α to exert its protective effects (Cappetta et al., 2016). PGC1α, in turn, regulates the expression of TFAM, which bridges nuclear and mitochondrial signals and induces mtDNA transcription (Vega et al., 2015). At the same time, TFAM, together with Top1mt, maintains the stability of mtDNA (Khiati et al., 2014). Anthracycline drugs also affect the expression of the p53 gene (Zhuang et al., 2012), increasing the expression of p53 protein in the cytoplasm, promoting its binding with mitochondrial protein Parkin, inhibiting the clearance of damaged mitochondria, leading to sustained oxidative stress in damaged mitochondria, and exacerbating apoptosis (Hoshino et al., 2013). P53 gene-deficient mice, when subjected to DOX treatment, show a significant increase in mitochondrial integrity in cardiac muscles and a reduction in apoptotic cardiac muscle cells. Therefore, p53 might be one of the reasons for the occurrence of acute cardiac dysfunction after DOX treatment (Zhu et al., 2009; Dhingra et al., 2014; Fang et al., 2019) (Figure 4).

### 3.4 Muscle fiber degradation and sarcoplasmic reticulum calcium homeostasis imbalance

Anthracycline drugs’ cardiac damage is related to muscle fiber degradation and disruption (Sawyer et al., 2002). In vertebrate skeletal muscle fibers, Titin plays a crucial role in the generation and stability of sarcomeres, serving as the assembling framework for muscle fibers (Granzier and Labeit, 2004). Calpain, a type of protease, can cause the degradation of Titin (Lim et al., 2004). When anthracycline drugs induce cardiac damage, it leads to the overexpression of Calpain within cardiac muscle cells, causing muscle fiber disruption and necrosis (Ali et al., 2010). Storr and others have demonstrated that endogenous inhibitor of Calpain, Calpastatin, can alleviate anthracycline drug-induced cardiac toxicity (Storr et al., 2011). It’s worth noting that Titin degradation is an early process of cardiac cell damage triggered by DOX (Lim et al., 2004). Previous research has found Titin fragments in the urine of anthracycline-treated patients, which holds diagnostic significance for cardiac injury, reflecting potential myocardial damage (Tanihata et al., 2019) or skeletal muscle damage (Misaka et al., 2019). Therefore, Titin fragments can serve as a biomarker for detecting early anthracycline-induced cardiac damage.

Treatment with anthracycline drugs can cause an imbalance in intracellular sarcoplasmic reticulum calcium homeostasis (Sala et al., 2020). In normal cardiac muscle cells, Ca^2+^ is mainly stored in the mitochondria, sarcoplasmic reticulum, and sarcolemma, playing a vital role in maintaining the excitation-contraction coupling of cardiac muscle cells. Anthracycline drugs can disrupt the membrane’s permeability, activate Ca^2+^ channels in the sarcoplasmic reticulum, increase the release of Ca^2+^ from the sarcoplasmic reticulum to the cytoplasm, rapidly increase intracellular free Ca^2+^ concentration, affecting cardiac electrical activity, resulting in various arrhythmias, known as sarcoplasmic reticulum calcium overload. During anthracycline drug treatment, Ca^2+^/calmodulin-dependent protein kinase II (CaMKII) causes changes in the calcium signaling pathway-related genes (Luo et al., 2013). Impaired calcium homeostasis leads to the generation of intracellular reactive ROS, and anthracycline drugs reduce sodium and calcium exchange in the sarcolemma (Caroni et al., 1981). This calcium overload results in mitochondrial damage, aggravated oxidative stress, structural damage, and cell apoptosis. Anthracycline drugs can also inhibit the gene expression of Ca^2+^-ATPase on the sarcolemma of cardiac muscle cells, affecting the biosynthesis of Ca^2+^-ATPase, reducing its activity, reducing the sarcolemma’s Ca^2+^ uptake capability, leading to reduced ATP production in mitochondria, disrupting cardiac energy metabolism, worsening cell damage, and even causing cardiac cell death.

Anthracycline drugs can also activate cardiac matrix metalloproteinase 2 (MMP2) (Chan et al., 2018). In normal cardiac muscle cells, MMP2 is present in the sarcomeres and the cell cytoskeleton. When oxidative stress occurs, MMP2 is activated and responsible for degrading specific proteins, including Troponin I (TNI) (Wang et al., 2002), Myosin Light Chain 1 (MYL1) (Sawicki et al., 2005), α-Actinin (Sung et al., 2007), and Titin (Ali et al., 2010). Therefore, transgenic mice overexpressing MMP2 show reduced muscle contractile function, sarcomere disruption, and myosin protein hydrolysis (Bergman et al., 2007). In anthracycline drug-induced cardiac toxicity models, the increase in MMP2 levels and activity is a primary cause of early intracellular Titin protein hydrolysis and extracellular matrix remodeling (Chan et al., 2021). These adverse reactions can be prevented by oral MMP2 inhibitors, demonstrating the potential benefits of MMP2 inhibitors in preventing anthracycline-induced cardiac toxicity (Chan et al., 2021).

### 3.5 Oxidative stress

**FIGURE 5:**
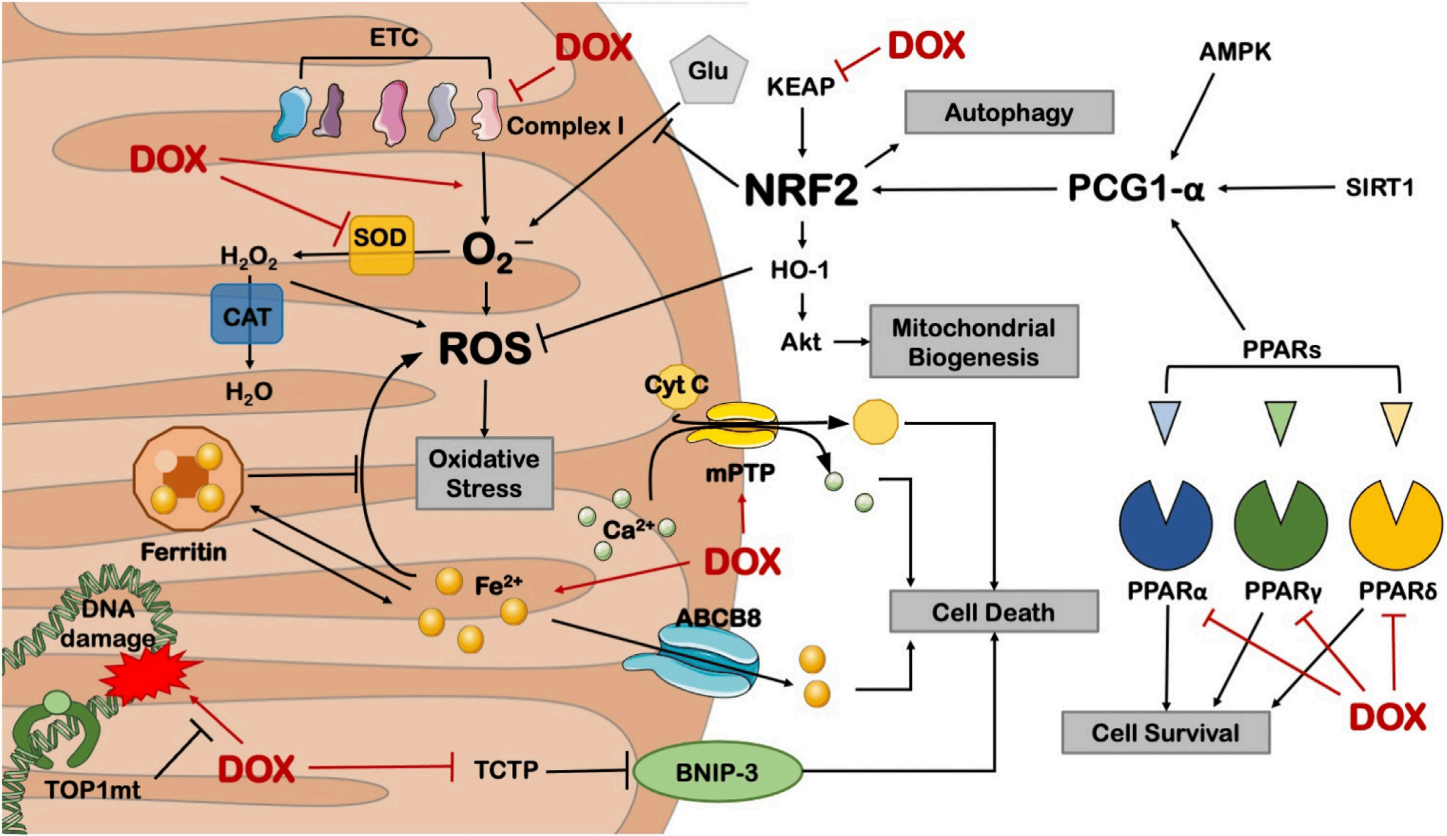
Anthracycline drugs can cause mitochondrial dysfunction by inhibiting the activity of mitochondrial transcription enzymes. These transcription enzymes mainly include Top1mt and mitochondrial transcription factors, and mitochondrial transcription factors include peroxisome proliferator-activated receptor-gamma coactivator-1α and 1β, Nuclear respiratory factor-1, mitochondrial transcription factor A (TFAM), and Tumor suppressor protein 53. Reproduced with permission from Schirone et al. (2022), licensed under CC BY 4.0.

Mitochondria are the primary targets of anthracycline drugs’ actions. Cardiac muscle cells are rich in mitochondria, with 35%–40% more mitochondria compared to other cells, making them susceptible to damage (Goffart et al., 2004). In normal signal transduction processes, low levels of ROS are generated to maintain intracellular signaling, but under many pathological conditions, ROS levels increase from low to high (Liou and Storz, 2010; Peoples et al., 2019; Songbo et al., 2019). When anthracycline drugs enter cardiac muscle cells, they generate a significant amount of ROS by Generation of large amounts of ROS by reducing redox cycling in electron transport chain complex I, leading to ATP synthesis impairment (Davies et al., 1983). After the substantial ROS generation within mitochondria, anthracycline drugs transform into semiquinone radicals. Semiquinone radicals readily react with oxygen to generate O^2-^, which can react with SOD to produce H_2_O_2_. Simultaneously, anthracycline drugs also produce a substantial amount of iron ions within cardiac muscle cells. Iron ions, serving as the electron donors for H_2_O_2_ in the Fenton reaction, lead to the generation of -OH, sustaining ROS production (Canzoneri and Oyelere, 2008). Generated ROS react with mitochondrial lipids, proteins, and nucleic acids, disrupting mitochondrial functions, including protein translation, expression, and inducing lipid peroxidation, generating a substantial amount of malondialdehyde (MDA). MDA is one of the major products of membrane lipid peroxidation, and its content is often used to indicate the degree of cellular membrane lipid peroxidation. Studies have found that if intracellular ROS levels continuously increase, it can worsen cellular membrane lipid peroxidation, leading to an increase in MDA content (Liu et al., 2018). Therefore, MDA content can also indirectly reflect the extent of myocardial damage (Eder and Arriaga, 2006). SOD is a metalloenzyme that plays a crucial role in the oxidative and antioxidative balance of the body, It catalyzes the disproportionation of O^2-^ to oxygen and hydrogen peroxide. (Henning et al., 2022).SOD can intercept the pathways of ROS-induced cell damage, timely repairing cell damage caused by ROS. As the primary scavenger of ROS within mitochondria, SOD plays an essential role in maintaining mitochondrial ROS levels and the stability of the mitochondrial environment (Bauer et al., 2014). In experiments on DOX-induced primary cardiac cell damage, DOX was found to increase intracellular MDA levels and reduce SOD activity, resulting in oxidative stress in cardiac cells (Dhingra et al., 2020). Wang et al. research showed that THP increased ROS levels in H9c2 cells and significantly elevated MDA content in rat serum while reducing SOD activity. RUT can reverse the oxidative stress damage caused by THP (Wang et al., 2018). In an experiment conducted by Aleixo and others on DOX-induced cardiac damage in rats, it was found that SOD activity decreased, MDA content increased in the cardiac tissues of model group rats compared to normal group rats, leading to the accumulation of a large amount of lipid peroxidation products in cardiac tissues, causing severe oxidative stress reactions (Marques-Aleixo et al., 2016) (Figure 5).

Research has also found that Nrf2, as a basic leucine zipper bZIP protein, can mitigate cell damage caused by ROS and electrophiles by regulating the expression of antioxidant proteins, keeping cells in a stable state, maintaining the dynamic balance of redox in the body, and counteracting oxidative damage. Nrf2 deficiency can exacerbate cardiac toxicity and cardiac dysfunction. In Nrf2 gene knockout (Nrf2^−/−^) mice, the adverse reactions of DOX cause cardiac muscle cell necrosis and cardiac dysfunction, which are related to oxidative stress, impaired autophagy, and increased accumulation of polyubiquitin aggregates (Li et al., 2014).

### 3.6 Cell apoptosis

**FIGURE 6:**
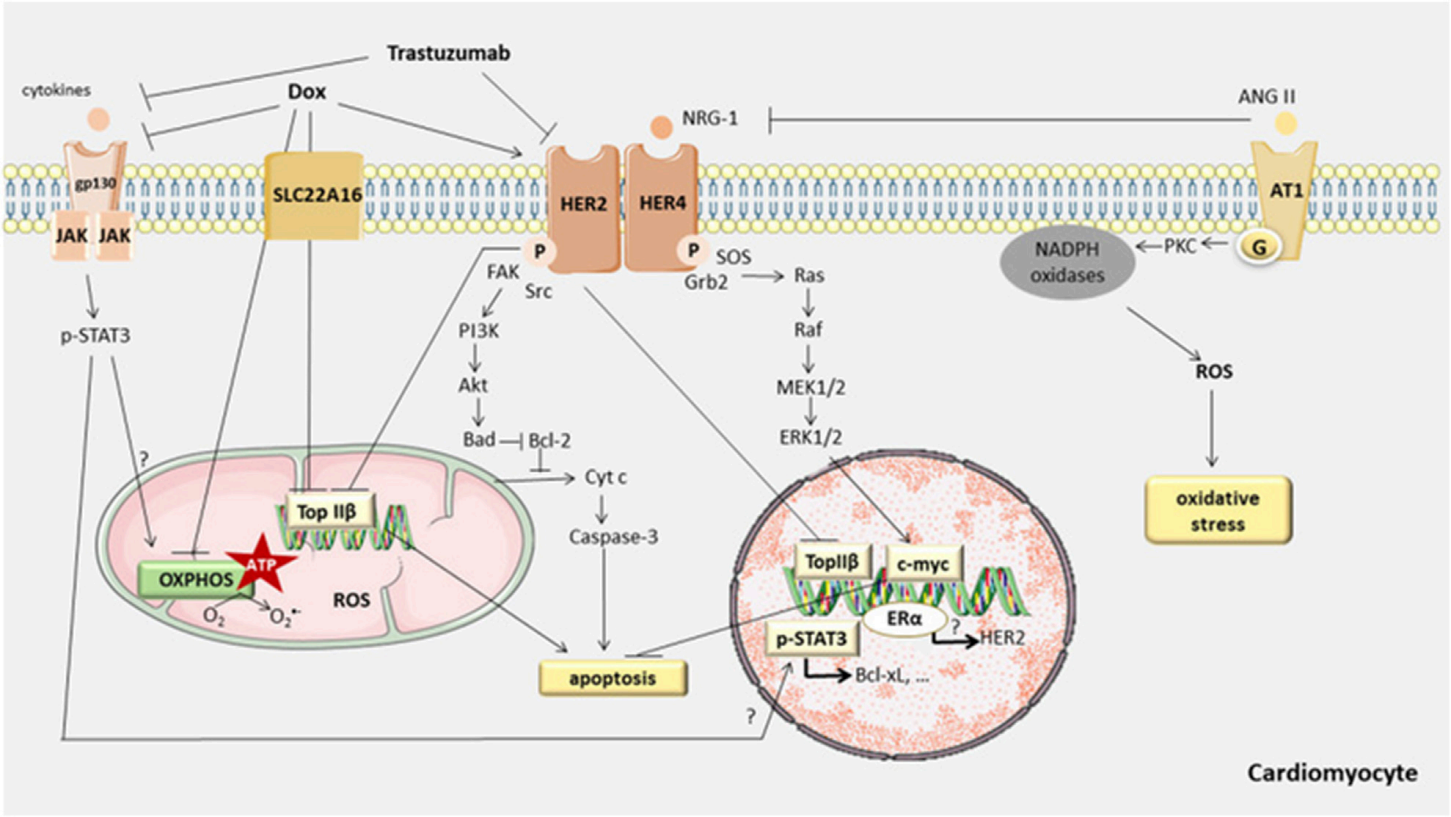
Anthracycline-induced cell apoptosis is mitochondria-mediated intrinsic signaling. When anthracycline drugs enter cardiac myocytes, they cause the release of Cyt c from the mitochondrial inner membrane, which then binds to Apaf-1 to form the apoptosome. The apoptosome activates the caspase family, leading to cell apoptosis. AKT, as a key factor in the apoptosis signaling pathway, can inhibit cell apoptosis by regulating the Bcl-2 family. In addition to the mitochondriamediated intrinsic signaling pathway, anthracycline drugs can also induce programmed necrosis and cell pyroptosis, resulting in damage to cardiac myocytes. Reproduced with permission from Anjos et al. (2021), Copyright 2021 Elsevier Inc. All rights reserved.

In the normal physiological state, the cardiomyocyte is a terminally differentiated, non-proliferative mature cell. Therefore, apoptosis of cardiac myocytes plays a crucial role in the cardiotoxicity induced by anthracyclines. Recent studies have confirmed that anthracycline drugs can induce apoptosis in cardiac myocytes, thereby damaging the myocardium. The primary pathway of anthracycline-induced cell apoptosis is mitochondria-mediated intrinsic signaling. When anthracycline drugs enter cardiac myocytes, they cause the release of Cyt c from the mitochondrial inner membrane, which then binds to Apaf-1 to form the apoptosome. The apoptosome activates the caspase family, leading to cell apoptosis. AKT, as a key factor in the apoptosis signaling pathway, can inhibit cell apoptosis by regulating the Bcl-2 family. In addition to the mitochondria-mediated intrinsic signaling pathway, anthracycline drugs can also induce programmed necrosis and cell pyroptosis, resulting in damage to cardiac myocytes (Figure 6).

#### 3.6.1 Mitochondria-mediated intrinsic pathway

Cell apoptosis (Apoptosis) is the primary mechanism of anthracycline anticancer activity and a major cause of non-cancer cell toxicity, especially in non-regenerative organs like the heart. Cell apoptosis primarily involves the regulation of two signaling pathways: the extrinsic pathway, which relies on cell surface “death” receptors, and the intrinsic pathway, which relies on mitochondria (Chowdhury et al., 2006). When oxidative stress or damage occurs within the cell, the mitochondria-mediated intrinsic signaling pathway is activated, leading to cell apoptosis. The mitochondria-mediated intrinsic cell apoptosis pathway primarily depends on the balance of pro-apoptotic and anti-apoptotic factors within the cell, which are members of the Bcl-2 superfamily. These factors control the permeability of the mitochondrial membrane (Skommer et al., 2007). The mitochondrial inner and outer membranes contain factors that can initiate the apoptotic cascade, such as Cyt c. When the permeability of the mitochondrial membrane is altered after anthracycline entry into the cell, Cyt c is released into the cytosol, where it can bind to Apaf-1 (Jourdain and Martinou, 2009), generating apoptotic vesicles, which can aggregate cysteoaspartic enzymes and activate cyst caspases through proteolytic cleavage, resulting in apoptotic cell death. (Soengas et al., 1999). The Bcl-2 family can be divided into three subtypes: Bcl-2-like anti-apoptotic factors, Bax-like pro-apoptotic factors, and BH3-only protein pro-apoptotic factors (Ferrans, 1978). Research has shown that serine/threonine kinase (AKT) can directly regulate the Bcl-2 family and indirectly regulate cell apoptosis by modulating apoptosis-related transcription factors. AKT, also known as protein kinase B (PKB), is a serine/threonine protein kinase with a molecular weight of approximately 60 kDa. AKT can be divided into three types (AKT1, AKT2, AKT3, or PKBα, PKBβ, and PKBγ), each with distinct functions but with some overlap. AKT1 is widely distributed in tissues, AKT2 is mainly found in muscle and fat cells, while AKT3 is expressed in the testes and the brain. AKT regulates various biological processes, including cell apoptosis, proliferation, growth, and glucose metabolism. The AKT signaling pathway is closely associated with the development of various diseases, such as malignancies, diabetes, and heart diseases (Franke et al., 1997; Linton et al., 2019; Uko et al., 2020).

Studies have reported that AKT inhibits cell apoptosis by primarily suppressing the activity of related apoptosis factors. BH3-only proteins (BOPs), as pro-apoptotic factors, can promote the binding of Bad to Bcl-2 or Bcl-xl. Bad regulates its binding to Bcl-2 through phosphorylation sites. When not stimulated by growth factors, Bad remains non-phosphorylated. Non-phosphorylated Bad consistently binds to Bcl-2, preventing Bcl-2 from exerting its anti-apoptotic function. When AKT is activated, it phosphorylates Bad at serine 136 (Nechushtan et al., 1999), causing Bad to dissociate from the Bcl-2 complex and bind to the scaffolding protein 14-3–3 (Masters et al., 2004). Binding of Bad to 14-three to three proteins initially isolates it from the mitochondrial outer membrane and prevents dephosphorylation of Bad, thus inhibiting its re-association with Bcl-2, which affects the cell-protective function of Bcl-2.

AKT can also directly regulate another member of the Bcl-2 family, Bax. Bax is a pro-apoptotic factor that can exert its function only when located in the mitochondrial outer membrane. In the absence of apoptosis stimuli, Bax is found in the cytoplasm. When cell apoptosis occurs, Bax undergoes conformational changes, enabling it to aggregate and insert into the mitochondrial outer membrane more easily (Gardai et al., 2004). Once inserted into the mitochondrial outer membrane, Bax forms oligomeric pores, allowing Cyt c and other pro-apoptotic factors to be released from the mitochondrial membrane. AKT phosphorylates Bax in a manner similar to Bad (Xin and Deng, 2005). AKT phosphorylates Bax at Ser184, inhibiting its conformational changes, thus preventing Bax from translocating to the mitochondrial membrane (Yamaguchi and Wang, 2001). This prevents the formation of oligomeric pores and the release of pro-apoptotic factors, thereby inhibiting cell apoptosis. Previous studies have found that in rat models of heart damage induced by anthracycline drugs, DOX induces apoptosis in cardiac tissues by inhibiting the AKT/Bcl-2 signaling pathway (Zhang Y. et al., 2020). Bai et al. made similar observations in a mouse model of DOX-induced cardiac damage, demonstrating that DOX can reduce p-AKT and Bcl-2/Bax protein expression, leading to the occurrence of cell apoptosis (Bai and Wang, 2019).

#### 3.6.2 Other pathways

AKT can also promote the expression of Fibronectin type III domain-containing protein 5 (FNDC5), which possesses certain cardioprotective properties. FNDC5 can inhibit the degradation of nuclear factor-erythroid2 related factor2 (Nrf2), mitigating anthracycline-induced cardiac damage (Zhang X. et al., 2020). Nrf2 plays a role in protecting myocardial cells by stabilizing the redox homeostasis and autophagic flux (Zhao et al., 2018).

In addition to apoptosis, anthracyclines induce other forms of cell death, including programmed necrosis (Necroptosis). Research has confirmed the association of necroptosis with the Ca^2+^/CaMKII pathway (Zhang T. et al., 2016) and the BH3 region proteins BOPs/Adenovirus E1B 19 kDa Interacting Protein3 (BNIP3) signaling pathway (Dhingra et al., 2014). However, inhibiting cell apoptosis or necroptosis only partially enhances myocardial cell survival (Zhang T. et al., 2016), indicating the involvement of other signaling pathways, such as pyroptosis (Meng et al., 2019). Pyroptosis results in cell swelling until the cell membrane ruptures, leading to the release of cellular contents and activating a strong inflammatory response.

## 4 Genetically driven mechanisms of anthracycline cardiotoxicity

**FIGURE 7:**
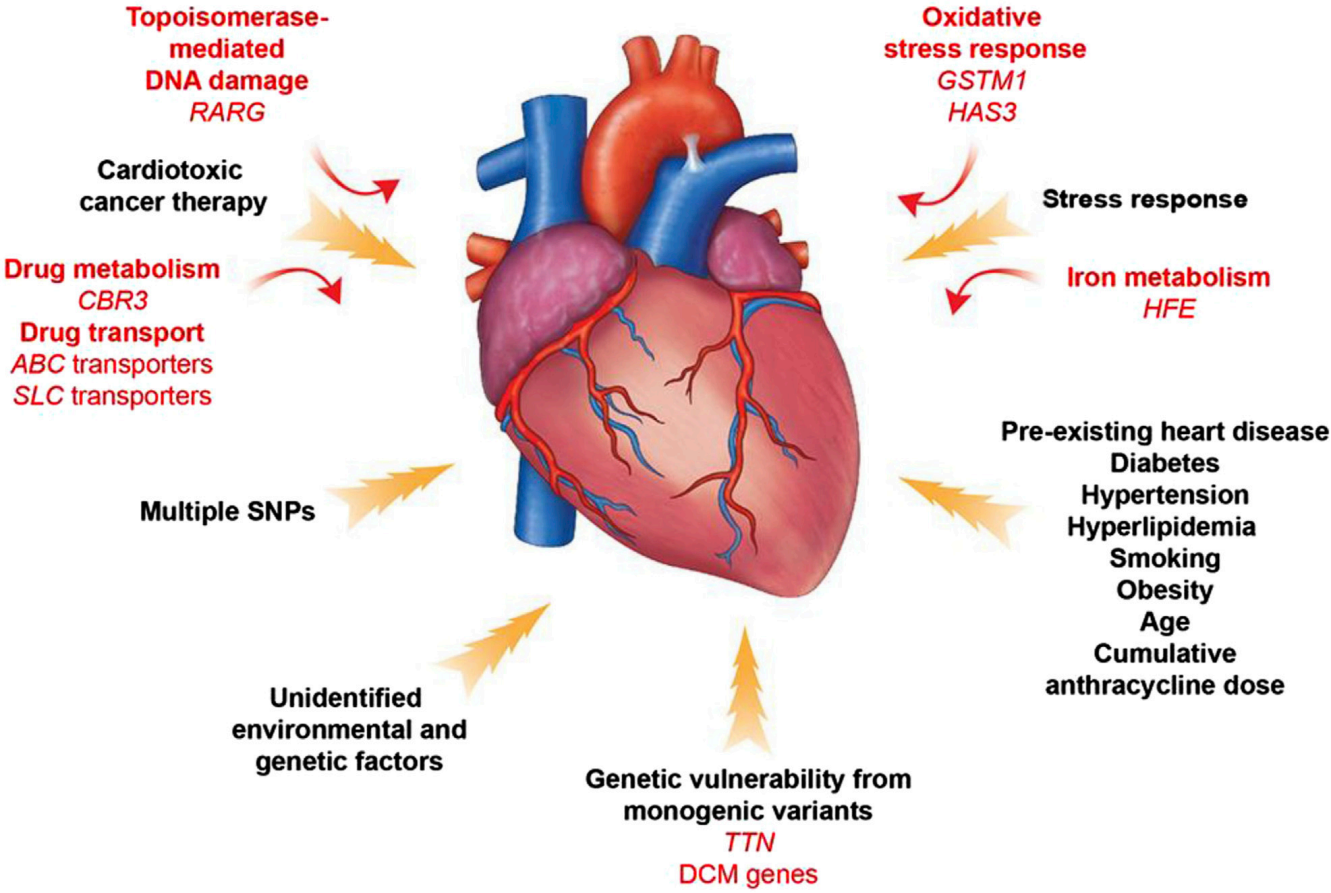
Contributors to Anthracycline -induced cardiovascular toxicity. A combination of clinical and genetic risk factors leads to increased risk of developing toxicity upon cancer therapy treatment. Elucidation of genetic contributors of cancer therapy-induced cardiovascular toxicity facilitates understanding of its molecular mechanism and development of its therapeutic strategies. Reproduced with permission from Kim et al. (2022), Copyright 2022 Elsevier Ltd. All rights reserved.

Identifying patients at high risk of anthracycline-induced cardiac injury using guidelines and predictive models remains extremely challenging. In addition, available parameters are not sufficient to explain why only some high-risk patients develop cardiotoxicity after treatment with antitumor drugs. The dose of chemotherapy with anthracyclines that results in toxic reactions varies significantly between patients. For example, some patients are able to tolerate a dose of 1,000 mg/m^2^ of adriamycin, whereas others develop acute cardiotoxicity at a dose of 200 mg/m^2^. These phenomena suggest that there may not be a “safe” anthracycline dose for all patients. This suggests that sensitivity to anthracyclines varies significantly among individuals and reveals the importance of genetic drivers of anthracycline-induced cardiotoxicity (Mulrooney et al., 2009) (Figure 7).

In a genome-wide unbiased chain-directed association study based on the HapMap project online CEU dataset, a total of 31,312 high-frequency single-nucleotide polymorphisms (SNPs) spanning over 1,278 genes were examined, as well as 86 lymphoblastoid cell lines susceptible to zorubicin. It was found that the heritability of the IC50 value for Zoerythromycin was 0.29. (Duan et al., 2007). Pathway analysis showed that phosphatidylinositol signaling system, GPI-anchored proteins, and axon guidance pathway were frequent in the list of candidate genes. This study proposes that the cardiotoxicity caused by Zoerythromycin may be influenced by many genes, with each gene having an impact at varying amounts.

In a large-scale candidate gene study involving 1,697 subjects, researchers chose genes related to the metabolism of reactive oxygen species, drug transport and metabolism, DNA repair, endothelial physiology, the renin-angiotensin-aldosterone system, muscle contraction and structure, inflammation, and cell cycle (Wojnowski et al., 2005). The genotypes of polymorphisms in 73 genes were determined in peripheral blood cells of individuals with non-Hodgkin’s lymphoma. These genotypes were then analyzed to determine if they were associated with acute and chronic cardiotoxicity. The authors concluded that anthracycline-induced cardiotoxicity was significantly associated with genetic polymorphisms in NAD(P)H oxidase and efflux transporter proteins (MRP1 and MRP2). An extensive review conducted a thorough analysis of 28 papers investigating the association between potential genes, analyzing a total of 84 genes and 147 single nucleotide polymorphisms (Leong et al., 2017). The presence of three risk alleles in the ABCC2, CyBA, and RAC2 genes was found to dramatically elevate the likelihood of developing anthracycline-induced cardiotoxicity.

Another experiment aimed to identify common variants that transmit disease risk in the general population. Researchers used a genotyping array to study patient DNA samples, which contained SNPs near candidate genes or whole genome SNPs. When using the latter approach, genome-wide association studies (GWAS) incorporate estimation by statistically analyzing the haplotypes in a genotyped reference panel to infer SNPs that are not directly genotyped. By far the most common group of genes associated with cardiovascular toxicity are involved in drug transport. The adenosine triphosphate-binding cassette transporter (ABC) proteins are dynamic cellular transporters for several medicines, including anthracyclines (Januchowski et al., 2014). Polymorphisms in specific genes, such as ABCB4, ABCC1, and ABCC2, have been linked to a higher likelihood of developing heart problems after receiving anthracycline treatments in both children and adults with blood-related and solid tumors (Wojnowski et al., 2005; Visscher et al., 2012; Armenian et al., 2013; Vulsteke et al., 2015).

In contrast, a genetic variant in ABCB1 appears to be cardioprotective (Hertz et al., 2016). Because this gene encodes an efflux transporter, a plausible explanatory model is that SNP increases drug clearance by cardiomyocytes. Gene variants in the soluble carriers (SLCs) gene family are also protective against anthracycline-induced cardiomyopathy. The genes involved include SLC22A7, SCL22A17, SLC10A2, and SLC28A3. Recently, Magdy et al. replicated the cardioprotective impact of the SLC28A3 locus in cardiomyocytes derived from human induced pluripotent stem cells. They also showed that the SLC-competitive inhibitor, desipramine, has a protective effect against doxorubicin-induced cardiotoxicity (Magdy et al., 2022). These transporters are found in cardiovascular cells and are responsible for transporting doxorubicin, nucleoside therapies, and other substances that occur naturally in the body.

Oxidative stress has a crucial role in the development of several cardiovascular disorders. Anthracycline metabolites also generate an excessive amount of reactive oxygen species and oxygen radicals. Given the understanding that these common signals may explain the clinical link between pre-existing cardiovascular disease and heightened susceptibility to harmful effects of cancer therapies, Wang et al. examined SNPs associated with 2,100 genes that have been classified as cardiovascular disease genes. These analyses identified a SNP in hyaluronan synthase 3 (HAS3) that was associated with high doses of anthracyclines treatment was significantly associated with cardiomyopathy (Wang et al., 2014). They found that HAS3 encodes an enzyme involved in the synthesis of hyaluronic acid, a constituent of the extracellular matrix, which acts as a scaffolding for the organization of cardiac cells, especially during remodeling after injury. Hyaluronic acid also has antioxidant properties that promote cardiac survival in the face of oxidative stress, which can lead to myocardial dysfunction (Jiang et al., 2011). Therefore, the HAS3 A/A genotype and associated lower hyaluronic acid levels may increase sensitivity to reactive oxygen species and increase the risk of myocardial infarction after anthracycline exposure.

Anthracyclines attach to iron and disrupt iron metabolism, while dexrazoxane, a compound derived from ethylenediaminetetraacetic acid, binds to iron and decreases the formation of anthracycline-iron complexes, hence decreasing the harmful effects of anthracyclines (Swain and Vici, 2004; Ichikawa et al., 2014). The significance of these biochemical interactions has been confirmed through genetic analysis. The HFE gene, responsible for encoding a cell-surface protein that controls the absorption of iron, undergoes mutations in individuals with hemochromatosis, a condition characterized by poor iron storage. A missense SNP in HFE (His63Asp) was significantly enriched in breast cancer patients treated with anthracyclines, who were more likely to develop cardiomyopathy compared to patients without cardiotoxicity patients who were more likely to develop cardiomyopathy. In addition, it was found that anthracyclines significantly increased iron accumulation in multiple organs in mutant mice lacking the HFE gene, leading to more severe mitochondrial damage and earlier death (Miranda et al., 2003).

## 5 Mechanisms of uptake and metabolism of anthracycline cardiotoxicity

The field of studying the effects of DOX on cardiac metabolism has not been fully explored. Recent metabolomics studies suggest that alterations in cardiac metabolism may play a key role in the development of DOX cardiotoxicity. Impaired oxidative phosphorylation and sustained active glycolysis are commonly observed during DOX treatment, which may impair the ability of cardiomyocytes to supply energy, ultimately leading to energy depletion. In addition, there is growing evidence that DOX cardiotoxicity may be associated with dysregulated insulin signaling and cardiac insulin resistance (Russo et al., 2021).

Chemotherapeutic drugs with cardiotoxic effects also disrupt intracellular systems responsible for regulating heart metabolism (Deidda et al., 2019). DOX specifically causes systemic insulin resistance, which is akin to type II diabetes, and increases levels of serum triglycerides and glucose (Arunachalam et al., 2013). Simultaneously, DOX induces a significant increase in glucose absorption by the heart. Cardiac metabolism is a complex mechanism and normally fatty acids (FA) are its main source of energy (Bertero and Maack, 2018). However, in response to pathological injury, the heart shifts to rely on glycolysis. While initially serving as a compensatory response, chronic dependence on glycolysis eventually results in maladaptation and energy depletion. This occurs when glycolysis and reduced mitochondrial function prevent cardiomyocytes from meeting their energy requirements (Stanley et al., 2005). Studies using animal models have demonstrated that cardiac insulin resistance and metabolic changes, such as reduced ability of mitochondria to metabolize glucose, lactate, and fatty acids, are early indicators of cardiac stress. Two of these antidiabetic drugs, metformin (MET) and empagliflozin (EMPA), have shown significant results as they not only lower blood glucose, but also prevent cardiometabolic diseases as well as DOX-induced cardiotoxicity (Zilinyi et al., 2018; Oh et al., 2019).

## 6 Existing and novel cardioprotective therapeutics and mechanisms

### 6.1 The cardioprotective effect of dexrazoxane on the cardiotoxicity of anthracycline drugs

Dexrazoxane is a substance that binds to iron and decreases the levels of reactive oxygen species. It has been proven to be a successful agent in protecting the heart in patients who are at a high risk and are receiving anthracycline therapy (Reichardt et al., 2018). The primary antitumor mechanisms of anthracyclines include inhibition of Top2, which leads to double-stranded DNA breaks, mitochondrial dysfunction, and increased ROS production (Bures et al., 2017). However, these mechanisms also lead to cardiomyocyte damage, which increases the risk of cardiotoxicity. The United States Food and Drug Administration (FDA) has implemented stringent guidelines to address the heart toxicity caused by dexrazoxane. These guidelines restrict the use of dexrazoxane to women with advanced metastatic breast cancer who have been treated with a minimum of 300 mg/m^2^ of doxorubicin or 540 mg/m^2^ of epirubicin (Swain et al., 1997; Ganatra et al., 2019). This is mainly because the use of dexrazoxane may increase the risk of infection, decrease the rate of anticancer response, and elevate the risk of secondary malignancies (Swain et al., 1997).

The cardioprotective effects of dexrazoxane are currently thought to include inhibition of DOX-Top2 complex formation, thereby preventing deleterious cardiomyocyte apoptosis (Bures et al., 2017). In addition, in a recent study, Yu et al. demonstrated that dexrazoxane was able to effectively ameliorate DOX-induced inflammation and necrosis by inhibiting the NF-kB and p38 MAPK pathways (Yu et al., 2020). Although the cardioprotective effect and clinical application of dexrazoxane still need to be further evaluated, it is currently believed that its protective mechanisms include cellular iron chelation, attenuation of cardiomyocyte apoptosis, iron apoptosis and necrosis (Swain et al., 1997; Bures et al., 2017; Reichardt et al., 2018; Yu et al., 2020). Therefore, as an adjuvant to anthracycline therapy, dexrazoxane has potential clinical value in high-risk patients, and its efficacy and safety need to be further verified in future studies.

### 6.2 The cardioprotective effect of β-blockers on the cardiotoxicity of anthracycline drugs

The use of β-blockers as prophylaxis prevents the development of systolic heart failure in female patients with breast cancer treated with anthracyclines (Kalay et al., 2006; Kaya et al., 2013; Nabati et al., 2017; Abuosa et al., 2018). Recently, Zhang et al. demonstrated through *in vitro* and *in vivo* studies that the β-blocker carvedilol (Carvedilol) attenuates DOX-induced inflammation, oxidative stress, and activation of pro-apoptotic pathways (Zhang et al., 2019). Specifically, carvedilol reduces the phosphorylation of the transcription factor nuclear factor-κB (NF-κB) (Zhang et al., 2019). Phosphorylation of NF-κB leads to its nuclear localization, which triggers the expression of pro-inflammatory cytokines, such as interleukin (IL) 1β, IL-18 and tumor necrosis factor-α (TNF-α) (Zhang et al., 2019). It was shown that carvedilol reduced the levels of phosphorylated NF-κB and its downstream inflammatory cytokines IL-1β, IL-18 and TNF-α in DOX-treated mice and cells (Zhang et al., 2019). Thus, β-blockers may exert cardioprotective effects by down-regulating inflammatory pathways.

In addition, DOX decreases NRF2, and carvedilol has been shown to partially reverse this decline (Zhang et al., 2019). Carvedilol reduced oxidative stress in DOX-treated mice by increasing the expression of NRF2 and its downstream genes, such as NQO1, HO1, and SOD1 (Zhang et al., 2019) Thus, carvedilol enhanced the antioxidant capacity of cardiomyocytes, enabling them to resist anthracycline-induced oxidative damage more effectively. In addition, β-blockers attenuate anthracycline-induced cardiomyocyte apoptosis (Spallarossa et al., 2004; Arozal et al., 2010; Zhang et al., 2019). Carvedilol was able to partially reverse the anthracycline-induced increase in pro-apoptotic protein Bax and activated caspases (Arozal et al., 2010; Zhang et al., 2019). Given that ROS production is the cause of BAX activation, it is theorized that the antioxidant properties of carvedilol may be linked to the decrease in cardiomyocyte apoptosis (Minotti et al., 2004) To summarize, β-blockers safeguard cardiomyocytes from anthracycline-induced cardiotoxicity by employing three mechanisms: inhibiting inflammation, boosting the antioxidant capacity of cardiomyocytes, and decreasing apoptosis.

### 6.3 The cardioprotective effect of renin angiotensin system blockers on the cardiotoxicity of anthracycline drugs

Angiotensin-converting enzyme (ACE) inhibitors are believed to be beneficial because they help decrease the generation of ROS and boost the activity of scavenging free radicals. Both of these processes are often disrupted in anthracycline therapy (Hiona et al., 2011; Abd El-Aziz et al., 2001; Chopra et al., 1989; al-Harbi, 1993). Hydrogen peroxide is a ROS that can induce programmed cell death, known as apoptosis, in cardiomyocytes (Hiona et al., 2011). Research has demonstrated that the ACE inhibitor enalapril decreases the occurrence of electrons flowing out of sequence, perhaps leading to a reduction in the generation of ROS. This mechanism may contribute to enalapril’s ability to protect the heart (Hiona et al., 2011). In addition, previous studies have shown that ACE inhibitors such as enalapril and captopril may act as free radical scavengers (Abd El-Aziz et al., 2001; Chopra et al., 1989; al-Harbi, 1993). Although it was initially thought that ACE inhibitors required sulfhydryl groups in order to scavenge free radicals, later studies have shown that ACE inhibitors lacking sulfhydryl groups, such as enalapril, still have the ability to scavenge free radicals (Mira et al., 1994). Finally, it has been shown that both enalapril and captopril increase the activity of the endogenous antioxidant enzyme SOD (de Cavanagh et al., 1997). Altogether, these effects would enhance the antioxidant defenses of cardiomyocytes, thereby reducing their vulnerability to anthracycline-induced oxidative stress.

ACE inhibitors reduce the amount of circulating angiotensin II (Ang II), whereas angiotensin receptor blockers (ARBs) act by preventing Ang II from binding to the AT1 receptor (Saleem et al., 2010). Activation of the AT1 receptor is associated with the upregulation of extracellular signal-regulated protein kinases (ERKs), which stimulate cardiomyocyte hypertrophy (Akazawa et al., 2013). In addition, blockade of AT1 receptors with ARBs leads to Ang II stimulation of AT2 receptors, which is associated with some of its cardioprotective effects (Schmieder, 2005). For example, the ARB Valsartan has been shown to reduce the expression of inflammatory cytokines, including monocyte chemotactic protein-1 (MCP-1), tumor necrosis factor-α, IL-6, and IL-1β (Schmieder, 2005). Thus, it may be postulated that the cardioprotective impact of ARBs is attributed to their capacity to inhibit cardiac remodeling and reduce inflammation linked to anthracycline-induced cardiotoxicity (Akazawa et al., 2013).

### 6.4 The cardioprotective effect of statins on the cardiotoxicity of anthracycline drugs

Statins, which are often referred to as HMG-CoA reductase inhibitors, are medications used largely to decrease cholesterol levels in individuals with atherosclerotic cardiovascular disease (Schachter, 2005). In the context of preventing anthracycline-induced cardiac insufficiency, RAS antagonists and β-blockers are frequently examined. However, a randomized controlled trial conducted by Nabati et al. (Nabati et al., 2019) revealed that statins have a protective effect in preventing chemotherapy-induced cardiotoxicity in breast cancer patients. Statins have little impact on hemodynamic indicators, specifically heart rate and blood pressure, making them potentially more manageable for people with these conditions.

Despite differences in the pharmacodynamics of the various statin drugs, they all reduce hepatocellular cholesterol synthesis by inhibiting HMG-CoA reductase, the rate-limiting step in the conversion of HMG-CoA to mevalonate (Schachter, 2005). Administration of statins reduces plasma concentrations of LDL, which causes atherosclerosis, while increasing concentrations of HDL, which has antioxidant, anti-inflammatory and antithrombotic effects (Henninger and Fritz, 2017). It has been suggested that statins exert their main effects by modulating Ras homolog (Rho) GTPase signaling (Henninger and Fritz, 2017). Studies have shown that Rho GTPase is able to reduce ROS levels by indirectly inhibiting the pro-oxidative NADPH system as well as modulating endothelial nitric oxide synthase (eNOS) (Schachter, 2005; Henninger and Fritz, 2017). In addition, *in vitro* studies have shown that cellular pretreatment with atorvastatin protects cardiomyocytes from DOX-induced apoptosis (Oh et al., 2020). DOX treatment activates the FOXO1 protein, which competitively inhibits the binding of STAT3 to Sp1 (Oh et al., 2020). The STAT3/Sp1 transcriptional complex is required for the expression of apoptosis inhibitory protein survivin (Oh et al., 2020). Pretreatment with statins is able to restore survivin in cardiomyocytes by inactivating FOXO1 through protein phosphorylation and inhibition of nuclear translocation (Oh et al., 2020). In addition to their cardioprotective effects, statins can sensitize tumor cells to certain chemotherapeutic agents while protecting cardiomyocytes, thereby increasing the anticancer response rate (Oh et al., 2020).

### 6.5 The cardioprotective effect of gut microbe-derived metabolites on the cardiotoxicity of anthracycline drugs

The role of microbial metabolites from food metabolism on the development of cardiometabolic diseases is now extensively acknowledged (Tremaroli and Bäckhed, 2012). The gut microbiota can function as an independent endocrine organ in the host body by secreting, degrading, or modifying chemicals through several metabolic pathways. These compounds have the ability to directly or indirectly impact organisms, which justifies the recognition of the gut microbiota as an endocrine organ. Short-chain fatty acids, such as acetic, propionic, and butyric acids, are key metabolites produced by the gut microbiota. These acids have been found to have significant impacts on heart function in animal models (Li et al., 2012).

The cardioprotective effect of butyric acid is mainly related to its epigenetic effect, as it is a potent inhibitor of histone deacetylase (HDAC), which is known to protect the heart from maladaptive hypertrophy and ischemic injury (Antos et al., 2003; Kong et al., 2006; Gallo et al., 2008; Granger et al., 2008). In addition, several studies by Raphaeli and colleagues revealed the dual activity of butyric acid and its precursor drugs, which on the one hand enhanced the antitumor activity of Dox in cancer models and on the other hand protected cardiomyocytes from Dox-induced cardiotoxicity (Rephaeli et al., 2007; Tarasenko et al., 2012; Moyal et al., 2018). Recently, the ability of *in vivo* oral administration of FBA, a new synthetic derivative of butyrate, to protect the heart from Dox cardiotoxicity and prevent mitochondrial dysfunction was demonstrated for the first time (Russo et al., 2019). Thus, the use of gut microbe-derived metabolites as nutritional supplements may represent a promising new approach for the treatment of Dox cardiotoxicity.

### 6.6 The cardioprotective effect of sports therapy on the cardiotoxicity of anthracycline drugs

Exercise has been acknowledged as a developing treatment for anthracycline-related cardiotoxicity. Research conducted on rodents has demonstrated that exercise is a potent stimulus that protects the heart and mitigates the harmful effects of DOX. A recent comprehensive analysis of two preclinical investigations found that physical activity interventions, such as forced treadmill running or voluntary free-wheel running, can reduce the damage to heart contractile function caused by DOX-induced fractional shortening (FS) injury (Ghignatti et al., 2021; Naaktgeboren et al., 2021). Exercise enhances cardiac function irrespective of the timing of the training in relation to DOX therapy. However, exercise conducted before DOX treatment is typically more successful in preserving cardiac contractility (Ghignatti et al., 2021). It is worth mentioning that even a brief period of aerobic exercise, lasting only 5 days, or a single exercise session of 60 min, might offer substantial advantages in reducing heart harm caused by DOX (Wonders et al., 2008; Lien et al., 2015). These findings offer compelling evidence to support the use of short- and long-term exercise interventions in the treatment of anthracycline cardiotoxicity.

Marques-Aleixo et al. conducted a study to examine the impact of 12 weeks of aerobic exercise, either through forced or voluntary wheel running, on the levels of protein carbonylation and lipid peroxidation produced by DOX in male rats (Marques-Aleixo et al., 2015). Exercise performed alongside DOX treatment was discovered to have a substantial impact on reducing cardiac protein carbonylation and lowering mitochondrial MDA levels. Chicco et al. found that an 8-week exercise regimen involving voluntary wheel running led to a notable decrease in left ventricular MDA levels in female rats (Chicco et al., 2005). In male rats, a 3-week exercise regimen or a single exercise session prior to DOX treatment also resulted in reduced myocardial MDA levels (Wonders et al., 2008; Shirinbayan and Roshan, 2012). The results indicate that engaging in exercise either at the same time or before receiving DOX treatment effectively reduces the negative effects of DOX on protein carbonylation and lipid peroxidation.

### 6.7 The role and mechanisms of iPSCs in anthracycline-induced cardiotoxicity

Induced human induced pluripotent stem cell-derived cardiomyocytes (hiPSC-CMs) are particularly important in cardiovascular research because other methods typically require invasive biopsies of human cardiac tissue, which are not sustainable in long-term culture. HiPSC-CMS not only overcome these limitations, but also mimic the physiological properties of *in vivo* cardiomyocytes, expressing most heart-specific ion channels and currents, and have a functional contractile apparatus. By analyzing their action potential morphology via membrane clamp, these cells can be further classified into atrial, ventricular, and sinus node subtypes (Burridge et al., 2016).

However, there are some limitations to using hiPSC-CMS. For example, they are considered immature and are closer to fetal cardiomyocytes in terms of structure, electrophysiological properties, genetic program and metabolism. They are structurally disordered in the myotome and lack T-tubules (Cao et al., 2008; Lundy et al., 2013). Due to differences in the *in vivo* environment and *in vitro* culture conditions, it is difficult for these cells to fully mimic the properties of adult cardiomyocytes. These shortcomings may complicate the interpretation of drug responses and make it difficult to predict translational effects in mature cells.

A future application of hiPSC-CMs may be the prediction of cardiotoxicity, a concept initially demonstrated by Burridge et al. in a study involving breast cancer patients treated with DOX. Although much work remains to be done in the preclinical application of hiPSC-CMs, the preliminary results are encouraging and may ultimately change the way drug screening is done, making it applicable to both groups and individuals. Currently, approaches to mitigating toxicity consist primarily of identifying known risk factors and comorbidities, followed by regular screening of patients by echocardiography, electrocardiography, angiography, and serum biomarkers (Manrique et al., 2017). Zhang et al. found that transplantation of iPSC-MSCs into a mouse model of anthracycline-induced cardiomyopathy retained more mitochondria in cardiac tissues and preserved the bioenergetics of cardiac tissues compared with bone marrow (BM)-MSCs. Unlike primary cardiomyocytes, which have limited availability and short lifespan in culture, cardiomyocytes derived via hiPSC-CMs can be easily generated and maintained *in vitro*. Models employing hiPSC can bridge the gap between cellular studies and animal models and clinical experiments, thus contributing to physiologically relevant mechanistic and target identification studies (Zhang Y. et al., 2016). Yang et al. monitored mitosis in hiPSC-CMs using the mt-Keima probe. The results showed that there were differences in basal mitotic rates in differentiated hiPSC-CMs. This result showed that cardiac mitosis was highly sensitive to the chemotherapeutic drug doxorubicin and hypoxia-induced cardiac ischemic conditions (Yang et al., 2022).

## 7 Animal experiments related to anthracycline-induced cardiotoxicity and its mechanism

For anthracycline chemotherapeutic drug-induced cardiotoxicity in humans, the guidelines in the U.S. and Europe differ in their criteria. The ESC2022 oncology cardiology guideline defines the lower limit of normal for the LVEF to be 50% (Michel et al., 2022), whereas in the U.S. it is 53% (Plana et al., 2014). Both guidelines emphasize a decrease in LVEF as an important marker of anthracycline cardiotoxicity.

The Chinese Guidelines for the Prevention and Treatment of Anthracycline Cardiotoxicity state that pharmacological cardiotoxicity refers to cardiac lesions, including arrhythmia, abnormal systolic/diastolic function, or even myocardial hypertrophy or cardiac enlargement, caused by toxicity of drugs to the myocardium and/or the cardiac electrical conduction system of patients receiving certain drug therapies. Antineoplastic drug cardiotoxicity is defined as cardiovascular injury with one or more of the following manifestations, excluding subclinical cardiovascular injury that occurs early in the course of chemotherapy/targeted drug use: (1) cardiomyopathy with decreased LVEF as evidenced by reduced overall function or markedly reduced septal motion; (2) symptoms related to CHF; (3) signs related to CHF, such as third heart sound gallop rhythm, tachycardia, or both; (4) a decrease in LVEF of at least 5% from baseline to an absolute value of <55%, accompanied by symptoms or signs of CHF; or a decrease in LVEF of at least 10% to an absolute value of <55%, not accompanied by symptoms or signs (Seidman et al., 2002), which can be diagnosed by the combination of clinical symptoms with electrocardiograms, echocardiograms, and isotope scans. At present, cardiotoxicity is mainly assessed according to the American New York Heart Association (NYHA) Classification and Evaluation of Cardiac Status or Adverse Events (CTC AE4.0) (Venturini et al., 1996; Marty et al., 2006).

Currently, strategies to reduce anthracycline cardiotoxicity are mainly based on the cardiotoxicity drug treatment should be adequately assessed before the risk of cardiotoxicity, as appropriate, appropriate adjustment of the drug dose or program, strengthen the monitoring of cardiac function, the use of other dosage forms (e.g., liposomal dosage forms), and so on. A large amount of high-level evidence-based medical evidence shows that DZR is the only drug that can effectively prevent anthracycline-induced cardiotoxicity (van Dalen et al., 2011), and has been included in clinical practice guidelines and widely used in the United States and the European Union. Meanwhile, the ACC/AHA Guidelines for the Diagnosis and Treatment of Chronic Heart Failure in Adults in the United States, anthracycline-induced heart failure/cardiomyopathy with tachyarrhythmia, in the treatment of anthracycline-induced heart failure, β-blockers are usually used for symptomatic treatment in the clinic. Other cardioprotective agents, including coenzyme Q10, leucovorin, N-acetylcysteine, antioxidants (e.g., VC and VE), and other iron chelators, may have some cardioprotective effect, but their use in the prevention and treatment of anthracycline-induced cardiotoxicity needs to be further investigated. Meta-analysis showed that coenzyme Q10, leucovorin, N-acetylcysteine, VC and VE had no significant cardioprotective effect on anthracycline chemotherapy, and that only dexrazoxane significantly benefited the patients, with a significantly lower incidence of heart failure (van Dalen et al., 2011). There are some other measures to reduce anthracycline cardiotoxicity. The chronic and delayed cardiotoxicity of anthracyclines is related to their cumulative dose, so limiting the cumulative dose of anthracyclines can reduce the incidence of cardiotoxicity (Von Hoff et al., 1979; Swain et al., 2003).

Studies have reported that cardiotoxicity of anthracyclines can also be reduced by continuous intravenous slow titration of anthracyclines instead of intravenous push, possibly through a mechanism that reduces the peak concentration of the drug. However, in a randomized trial it was found that 48 h of continuous infusion did not result in better cardioprotection than intravenous push (1 h injection) (Lipshultz et al., 2002). Therefore, whether changing the method of administration is a good way to prevent anthracycline cardiotoxicity still needs to be thoroughly investigated. In addition, the use of liposomal anthracyclines has the potential to reduce the incidence of anthracycline cardiotoxicity. Liposomal anthracyclines currently used in clinical practice include liposomal adriamycin and liposomal flexerodine. Polyethylene glycol liposomal adriamycin has a longer half-life because it is not phagocytosed by macrophages and monocytes (Gabizon and Martin, 1997), and the drug has a reduced concentration of drug distribution in the myocardium, which decreases the tendency of toxins to accumulate in the myocardial cells, and therefore reduces the cardiotoxicity and improves the safety relative to the traditional adriamycin (Barry et al., 2007).

Now, studies on animal models of antitumor therapy-induced cardiotoxicity have made remarkable progress in revealing the relevant mechanisms. For example, using a mouse model of adriamycin cardiotoxicity, Fang et al. identified iron-induced cell death as a critical mechanism underlying adriamycin cardiotoxicity, presenting a novel approach for preventing and treating adriamycin-induced cardiomyopathy (Fang et al., 2019). Zhang et al. demonstrated that DNA topoisomerase IIB (TOP2B) is a significant target of adriamycin cardiotoxicity, utilizing a mouse model to support their findings (Zhang et al., 2012). The identification of these mechanisms and targets has established a robust basis for the development of relevant therapeutic drugs (Telli et al., 2007).

Most of the studies on preventive and therapeutic drugs for cardiotoxicity prevention and treatment of antitumor therapy recommended by various guidelines have used animal models, such as statins, ACEIs, and ARBs. Li et al. used a mouse model of adriamycin cardiotoxicity to discover that statins significantly ameliorated cardiomyocyte damage in mice through antiapoptosis, proposing that statins could be used as a protective agent against DOX-induced cardiotoxicity (Li et al., 2006).

Additionally, animal models have been instrumental in studying the prevention and treatment of cardiotoxicity caused by new antitumor therapies. For instance, Li et al. discovered that thrombopoietin (TPO) significantly reduced cardiotoxicity via antioxidant and anti-inflammatory mechanisms using a mouse model of doxorubicin cardiotoxicity, presenting a novel therapeutic option (Li et al., 2006). Milano et al. employed a rat model to investigate cardiotoxicity induced by the combination of doxorubicin and trastuzumab, finding that miR146a-5p-mediated human CPC exosome mitigated doxorubicin-induced oxidative stress in the heart (Milano et al., 2020).

However, there are still some problems in applying animals for relevant studies. The first problem is the type of injection used to replicate the relevant animal model. In constructing animal models for DOX cardiotoxicity, researchers have primarily used mice, rats, rabbits, dogs, and zebrafish, administering DOX at doses ranging from 5 to 45 mg/kg either intraperitoneally or intravenously via the tail vein. Each administration method has its pros and cons. Intraperitoneal injection, though straightforward, may cause peritoneal injury, elevate the risk of noncardiac death, and hinder drug absorption, potentially impacting experimental outcomes. Conversely, tail vein injection, while circumventing these issues, poses operational challenges and raises the risk of phlebitis and tail necrosis (Li DL. et al., 2016).

The second problem is that there are no clear objective criteria or reference values in the relevant literature to uniformly describe the cardiotoxic response in animal models. The review by Nikolaos et al. summarized the range of echocardiographic indices of anthracycline cardiotoxicity in rats as an experimental model, focusing on the assessment of two major echocardiographic indices, namely, ejection fraction (EF) and fractional shortening (FS). An in-depth retrospective analysis did not reveal differences in EF and FS values decreased by anthracycline administration in different strains of rats, and acute and chronic anthracycline cardiotoxicity models were equally potent in inducing cardiotoxicity (Georgiadis et al., 2020). However, there may be large individual differences in single EF and FS indices. It has been suggested that a range of percent suppression of EF and FS reduces intra-individual variability and more effectively identifies early cardiotoxicity. Thomas et al. concluded that global myocardial strain (GLS) provides a more reliable and reproducible method of assessing overall cardiac function, and because the confidence interval for GLS is smaller than that for EF, GLS may detect changes in myocardial function earlier. Observational studies have shown that although absolute measures of GLS at baseline and during treatment are predictive of anthracycline cardiotoxicity risk, the most reliable method is to assess changes in GLS with treatment-a meaningful relative change of 10%–15% is considered significant. This study implies that GLS is a window into subsequent changes in EF, which can effectively provide an opportunity for early intervention and thus hopefully more effective intervention (Wang et al., 2021). Jolanda et al. also used Longitudinal strain (LS) and Circumferential strain (CS) changes in experiments in dox mice as an indicators of injury (Sabatino et al., 2020).

In addition to conducting more large-scale, prospective, multi-center studies in the future to better assess the risk factors of cardiac injury in such patients, and even to prevent the occurrence of cardiac injury in patients with tumors, so as to make the intervention more comprehensive and targeted; it is also necessary to standardize the indexes related to the relevant animal models, so as to carry out the follow-up study to achieve real and credible results.

## 8 Conclusion

This review provides an overview of the different types of cardiac damage induced by anthracycline-class drugs and delves into the molecular mechanisms behind these injuries. Cardiac damage primarily involves alterations in myocardial cell function and pathological cell death, encompassing mitochondrial dysfunction, topoisomerase inhibition, disruptions in iron ion metabolism, myofibril degradation, and oxidative stress. Current literature suggests that anthracyclines further affect heart health by binding to DNA topoisomerases and inserting themselves into DNA, leading to DNA cleavage. Additionally, they are associated with mitochondrial and myofibril degradation, thereby increasing the risk of apoptosis. Mechanisms of uptake and transport in anthracycline-induced cardiotoxicity are emphasized, as well as the role and breakthroughs of iPSC in cardiotoxicity studies. Selected novel cardioprotective therapies and mechanisms are updated. Mechanisms and protective strategies related to anthracycline cardiotoxicity in animal experiments are investigated, and the definition of drug injury in humans and animal models is discussed according to relevant guidelines. Understanding these molecular mechanisms is of paramount importance in mitigating anthracycline-induced cardiac toxicity and guiding the development of safer approaches in cancer treatment.

## Figures and Tables

**Table:** 

**DAU**	Daunorubicin
**DOX**	Doxorubicin
**IVC**	inferior vena cava
**CRRT**	continuous renal replacement therapy
**CT**	computed tomography
**ICU**	intensive care unit
**ALT**	alanine transaminase
**AST**	aspartate transaminase
**TBIL**	total bilirubin
**DBIL**	direct bilirubin
**THP**	Pirarubicin
**IDU**	Idarubicin
**EPI**	Epirubicin
**DZR**	Dexrazoxane
**LVEF**	left ventricular ejection fraction
**Top**	Topoisomerase
**IRP1**	Iron Regulatory Protein 1
**TF**	Transferrin receptor
**ABCB8**	ATP Binding Cassette Transporter B8
**PPARα**	peroxisome proliferators activated receptor α
	Bcl-2-like proteins
**tBid**	truncated bid
**Apaf-1**	Apoptotic protease activating facter-1
**CARD**	Caspase recruitment domain
**NRF1**	Nuclear respiratory factor-1
**TFAM**	mitochondrial transcription factor A
**p53**	Tumor suppressor protein53
**SIRT1**	sirtuin1
**MMP2**	matrix metallo proteinase2
**TNI**	TroponinI
**MYL1**	Myosin Light Chain 1
**MDA**	malondialdehyde
**Nrf2**	nuclear factor-erythroid2 related factor2
**BOPs**	BH3-onlyproteins
**SNPs**	single-nucleotide polymorphisms
**GWAS**	genome-wide association studies
**HAS3**	hyaluronan synthase 3
**FA**	fatty acids
**MET**	Metformin
**EMPA**	empagliflozin
**FDA**	Food and Drug Administration
**ACE**	Angiotensin-converting enzyme
**Ang II**	angiotensin II
**MCP-1**	monocyte chemotactic protein-1
**ARBs**	angiotensin receptor blockers
**HDAC**	histone deacetylase
**MDA**	malondialdehyde
**hiPSC-CMs**	Human induced pluripotent stemcell-derived cardiomyocytes
**GLS**	global myocardial strain

## References

[B1] Abd El-Aziz M. A. Othman A. I. Amer M. El-Missiry M. A. (2001). Potential protective role of angiotensin-converting enzyme inhibitors captopril and enalapril against adriamycin-induced acute cardiac and hepatic toxicity in rats. J. Appl. Toxicol. 21, 469–473. 10.1002/jat.782 11746193

[B2] Abuosa A. M. Elshiekh A. H. Qureshi K. Abrar M. B. Kholeif M. A. Kinsara A. J. (2018). Prophylactic use of carvedilol to prevent ventricular dysfunction in patients with cancer treated with doxorubicin. Indian Heart J. 70 (Suppl. 3), S96-S100–s100. 10.1016/j.ihj.2018.06.011 30595329 PMC6310701

[B3] Akazawa H. Yabumoto C. Yano M. Kudo-Sakamoto Y. Komuro I. (2013). ARB and cardioprotection. Cardiovasc. Drugs Ther. 27, 155–160. 10.1007/s10557-012-6392-2 22538956

[B4] al-Harbi M. M. (1993). Effect of captopril on the cytological and biochemical changes induced by adriamycin. Food Chem. Toxicol. 31, 209–212. 10.1016/0278-6915(93)90095-g 8473005

[B5] Ali M. A. Cho Wj Fau - Hudson B. Hudson B. F.-K. Z. Granzier H. Schulz R. (2010). Titin is a target of matrix metalloproteinase-2: implications in myocardial ischemia/reperfusion injury. Circulation 122 (20), 2039–2047. 10.1161/CIRCULATIONAHA.109.930222 21041693 PMC3057897

[B206] Anjos M. Fontes-Oliveira M. Costa V. M. Santos M. Ferreira R. (2021). An update of the molecular mechanisms underlying doxorubicin plus trastuzumab induced cardiotoxicity. Life Sci. 280, 119760. 10.1016/j.lfs.2021.119760 34166713

[B6] Antos C. L. McKinsey T. A. Dreitz M. Hollingsworth L. M. Zhang C. L. Schreiber K. (2003). Dose-dependent blockade to cardiomyocyte hypertrophy by histone deacetylase inhibitors. J. Biol. Chem. 278, 28930–28937. 10.1074/jbc.M303113200 12761226

[B7] Arcamone F. Fau - Cassinelli G. Cassinelli G. Fau - Fantini G. Fantini G. Fau - Grein A. (1969). Adriamycin, 14-hydroxydaunomycin, a new antitumor antibiotic from S. peucetius var. caesius. Repr. Biotechnol. Bioeng. 6 (6)), 1101–1110. 10.1002/bit.260110607 5365804

[B8] Arif T. Fau - Vasilkovsky L. Vasilkovsky L. Fau - Refaely Y. Refaely Y. Fau - Konson A. (2017). Silencing VDAC1 expression by siRNA inhibits cancer cell proliferation and tumor growth *in vivo* . Mol. Ther. Nucleic Acids 15 (8), 493–508. 10.1016/j.omtn.2017.08.008 28918049 PMC6114110

[B9] Armenian S. H. Ding Y. Mills G. Sun C. Venkataraman K. Wong F. L. (2013). Genetic susceptibility to anthracycline-related congestive heart failure in survivors of haematopoietic cell transplantation. Br. J. Haematol. 163, 205–213. 10.1111/bjh.12516 23927520 PMC3795883

[B10] Arozal W. Watanabe K. Veeraveedu P. T. Ma M. Thandavarayan R. A. Sukumaran V. (2010). Protective effect of carvedilol on daunorubicin-induced cardiotoxicity and nephrotoxicity in rats. Toxicology 274, 18–26. 10.1016/j.tox.2010.05.003 20452391

[B11] Arunachalam S. Tirupathi Pichiah P. B. Achiraman S. (2013). Doxorubicin treatment inhibits PPARγ and may induce lipotoxicity by mimicking a type 2 diabetes-like condition in rodent models. FEBS Lett. 587, 105–110. 10.1016/j.febslet.2012.11.019 23219922

[B12] Bai Z. A.-O. Wang Z. (2019). Genistein protects against doxorubicin-induced cardiotoxicity through Nrf-2/HO-1 signaling in mice model. Environ. Toxicol. 34 (5), 645–651. 10.1002/tox.22730 30734460

[B13] Bansal N. Adams M. J. Ganatra S. Colan S. D. Aggarwal S. Steiner R. (2019). Strategies to prevent anthracycline-induced cardiotoxicity in cancer survivors. Cardiooncology 2 (5), 18. 10.1186/s40959-019-0054-5 32154024 PMC7048046

[B14] Barry E. Alvarez Ja Fau - Scully R. E. Scully Re Fau - Miller T. L. Lipshultz S. E. (2007). Anthracycline-induced cardiotoxicity: course, pathophysiology, prevention and management. Expert Opin. Pharmacother. 8 (8), 1039–1058. 10.1517/14656566.8.8.1039 17516870

[B15] Bauer G. Bereswill S. Aichele P. Glocker E. (2014). *Helicobacter pylori* protects oncogenically transformed cells from reactive oxygen species-mediated intercellular induction of apoptosis. Carcinogenesis 35 (7), 1582–1591. 10.1093/carcin/bgu074 24662971

[B16] Benz R. Maier E. Fau - Thinnes F. P. Thinnes Fp Fau - Götz H. Hilschmann N. (1992). Studies on human porin. VII. The channel properties of the human B-lymphocyte membrane-derived “Porin 31HL” are similar to those of mitochondrial porins. Biol. Chem. Hoppe Seyler 373 (6), 295–303. 10.1515/bchm3.1992.373.1.295 1381184

[B17] Bergman M. R. Teerlink Jr Fau - Mahimkar R. Mahimkar R. Fau - Li L. Nguyen A. (2007). Cardiac matrix metalloproteinase-2 expression independently induces marked ventricular remodeling and systolic dysfunction. Am. J. Physiol. Heart Circ. Physiol. 292 (4), 1847–1860. 10.1152/ajpheart.00434.2006 17158653

[B18] Berry G. J. Jorden M. (2005). Pathology of radiation and anthracycline cardiotoxicity. Pediatr. Blood Cancer 44 (7), 630–637. 10.1002/pbc.20346 15825155

[B19] Bertero E. Maack C. (2018). Metabolic remodelling in heart failure. Nat. Rev. Cardiol. 15, 457–470. 10.1038/s41569-018-0044-6 29915254

[B20] Bures J. Jirkovska A. Sestak V. Jansova H. Karabanovich G. Roh J. (2017). Investigation of novel dexrazoxane analogue JR-311 shows significant cardioprotective effects through topoisomerase IIbeta but not its iron chelating metabolite. Toxicology 392, 1–10. 10.1016/j.tox.2017.09.012 28941780

[B21] Burridge P. W. Li Y. F. Matsa E. Wu H. Ong S. G. Sharma A. (2016). Human induced pluripotent stem cell-derived cardiomyocytes recapitulate the predilection of breast cancer patients to doxorubicin-induced cardiotoxicity. Nat. Med. 22, 547–556. 10.1038/nm.4087 27089514 PMC5086256

[B22] Buttke T. M. Sandstrom P. A. (1994). Oxidative stress as a mediator of apoptosis. Immunol. Today 15 (1), 7–10. 10.1016/0167-5699(94)90018-3 8136014

[B23] Canzoneri J. C. Oyelere A. K. (2008). Interaction of anthracyclines with iron responsive element mRNAs. Nucleic Acids Res. 36 (21), 6825–6834. 10.1093/nar/gkn774 18953029 PMC2588532

[B24] Cao F. Wagner R. A. Wilson K. D. Xie X. Fu J. D. Drukker M. (2008). Transcriptional and functional profiling of human embryonic stem cell-derived cardiomyocytes. PloS one 3, e3474. 10.1371/journal.pone.0003474 18941512 PMC2565131

[B25] Cappetta D. Esposito G. Piegari E. Russo R. Ciuffreda L. P. Rivellino A. (2016). SIRT1 activation attenuates diastolic dysfunction by reducing cardiac fibrosis in a model of anthracycline cardiomyopathy. Int. J. Cardiol. 205 (15), 99–110. 10.1016/j.ijcard.2015.12.008 26730840

[B26] Cardinale D. Colombo A. Bacchiani G. Tedeschi I. Meroni C. A. Veglia F. (2015). Early detection of anthracycline cardiotoxicity and improvement with heart failure therapy. Circulation 131 (22), 1981–1988. 10.1161/CIRCULATIONAHA.114.013777 25948538

[B27] Caroni P. Fau - Villani F. Villani F. Fau - Carafoli E. Carafoli E. (1981). The cardiotoxic antibiotic doxorubicin inhibits the Na+/Ca2+ exchange of dog heart sarcolemmal vesicles. FEBS Lett. 130 (2), 184–186. 10.1016/0014-5793(81)81115-5 6945186

[B207] Carvalho F. S. Burgeiro A. Garcia R. Moreno A. J. Carvalho R. A. Oliveira P. J. (2014). Doxorubicin-induced cardiotoxicity: from bioenergetic failure and cell death to cardiomyopathy. Med. Res. Rev. 34, 106–135. 10.1002/med.21280 23494977

[B28] Chan B. Y. H. Roczkowsky A. Cho W. J. Poirier M. Sergi C. Keschrumrus V. (2021). MMP inhibitors attenuate doxorubicin cardiotoxicity by preventing intracellular and extracellular matrix remodelling. Cardiovasc Res. 117 (1), 188–200. 10.1093/cvr/cvaa017 31995179 PMC7797218

[B29] Chan B. Y. H. Roczkowsky A. Moser N. Poirier M. Hughes B. G. Ilarraza R. (2018). Doxorubicin induces *de novo* expression of N-terminal-truncated matrix metalloproteinase-2 in cardiac myocytes. Can. J. Physiol. Pharmacol. 96 (12), 1238–1245. 10.1139/cjpp-2018-0275 30308129

[B30] Chicco A. J. Schneider C. M. Hayward R. (2005). Voluntary exercise protects against acute doxorubicin cardiotoxicity in the isolated perfused rat heart. Am. J. physiology Regul. Integr. Comp. Physiology 289, R424–r431. 10.1152/ajpregu.00636.2004 15845878

[B31] Chopra M. Scott N. McMurray J. McLay J. Bridges A. Smith W. E. (1989). Captopril: a free radical scavenger. Br. J. Clin. Pharmacol. 27, 396–399. 10.1111/j.1365-2125.1989.tb05384.x 2655686 PMC1379842

[B32] Chowdhury I. Tharakan B. Fau - Bhat G. K. Bhat G. K. (2006). Current concepts in apoptosis: the physiological suicide program revisited. Cell Mol. Biol. Lett. 11 (4), 506–525. 10.2478/s11658-006-0041-3 16977376 PMC6275981

[B33] Classen S. Olland S. Fau - Berger J. M. Berger J. M. (2003). Structure of the topoisomerase II ATPase region and its mechanism of inhibition by the chemotherapeutic agent ICRF-187. Proc. Natl. Acad. Sci. 100 (19), 10629–10634. 10.1073/pnas.1832879100 12963818 PMC196855

[B34] Clémençon B. Babot M. Fau - Trézéguet V. Trézéguet V. (2013). The mitochondrial ADP/ATP carrier (SLC25 family): pathological implications of its dysfunction. Mol. Asp. Med. 34 (2), 485–493. 10.1016/j.mam.2012.05.006 23506884

[B208] Corremans R. Adão R. De Keulenaer G. W. Leite-Moreira A. F. Brás-Silva C. (2019). Update on pathophysiology and preventive strategies of anthracycline-induced cardiotoxicity. Clin. Exp. Pharmacol. Physiol. 46, 204–215. 10.1111/1440-1681.13036 30244497

[B35] Davies Kj Fau - Doroshow J. H. Doroshow Jh Fau - Hochstein P. Hochstein P. (1983). Mitochondrial NADH dehydrogenase-catalyzed oxygen radical production by adriamycin, and the relative inactivity of 5-iminodaunorubicin. FEBS Lett. 153 (1), 227–230. 10.1016/0014-5793(83)80153-7 6298008

[B36] de Cavanagh E. M. Fraga C. G. Ferder L. Inserra F. (1997). Enalapril and captopril enhance antioxidant defenses in mouse tissues. Am. J. physiology 272, R514–R518. 10.1152/ajpregu.1997.272.2.R514 9124472

[B37] Deidda M. A.-O. Mercurio V. Cuomo A. A.-O. Noto A. Mercuro G. Cadeddu Dessalvi C. (2019). Metabolomic perspectives in antiblastic cardiotoxicity and cardioprotection. Int. J. Mol. Sci. 20 (19), 4928. 10.3390/ijms20194928 31590338 PMC6801977

[B38] Delbridge A. R. Grabow S. Strasser A. Vaux D. L. (2011). Thirty years of BCL-2: translating cell death discoveries into novel cancer therapies. FEBS Lett. 585 (4), 677–682. 10.1038/nrc.2015.17 26822577

[B39] Delbridge A. R. Grabow S. Strasser A. Vaux D. L. (2016). Thirty years of BCL-2: translating cell death discoveries into novel cancer therapies. Nat. Rev. Cancer 16 (2), 99–109. 10.1038/nrc.2015.17 26822577

[B40] Dhingra R. Guberman M. Rabinovich-Nikitin I. Gerstein J. Margulets V. Gang H. (2020). Impaired NF-κB signalling underlies cyclophilin D-mediated mitochondrial permeability transition pore opening in doxorubicin cardiomyopathy. Cardiovasc Res. 116 (6), 1161–1174. 10.1093/cvr/cvz240 31566215 PMC7177490

[B41] Dhingra R. Margulets V. Chowdhury S. R. Thliveris J. Jassal D. Fernyhough P. (2014). Bnip3 mediates doxorubicin-induced cardiac myocyte necrosis and mortality through changes in mitochondrial signaling. Proc. Natl. Acad. Sci. 111 (51), 5537–5544. 10.1073/pnas.1414665111 25489073 PMC4280597

[B42] Di Noia M. A. Todisco S. Cirigliano A. Rinaldi T. Agrimi G. Iacobazzi V. (2014). The human SLC25A33 and SLC25A36 genes of solute carrier family 25 encode two mitochondrial pyrimidine nucleotide transporters. J. Biol. Chem. 289 (48), 3137–48. 10.1074/jbc.M114.610808 25320081 PMC4246075

[B43] Doroshow J. H. Davies K. J. (1986). Redox cycling of anthracyclines by cardiac mitochondria. II. Formation of superoxide anion, hydrogen peroxide, and hydroxyl radical. J. Biol. Chem. 261 (7), 3068–3074. 10.1016/s0021-9258(17)35747-2 3005279

[B44] Duan S. Bleibel W. K. Huang R. S. Shukla S. J. Wu X. Badner J. A. (2007). Mapping genes that contribute to daunorubicin-induced cytotoxicity. Cancer Res. 67, 5425–5433. 10.1158/0008-5472.CAN-06-4431 17545624 PMC2735868

[B45] Eder A. R. Arriaga E. A. (2006). Capillary electrophoresis monitors enhancement in subcellular reactive oxygen species production upon treatment with doxorubicin. Chem. Res. Toxicol. 19 (9), 1151–1159. 10.1021/tx060083i 16978019 PMC2626132

[B46] Eliseev R. A. Malecki J. Lester T. Zhang Y. Humphrey J. Gunter T. E. (2009). Cyclophilin D interacts with Bcl2 and exerts an anti-apoptotic effect. J. Biol. Chem. 284 (15), 9692–9699. 10.1074/jbc.M808750200 19228691 PMC2665090

[B47] Fang X. A.-O. Wang H. A.-O. Han D. Xie E. Yang X. Wei J. (2019). Ferroptosis as a target for protection against cardiomyopathy. Proc. Natl. Acad. Sci. U. S. A. 116 (7), 2672–2680. 10.1073/pnas.1821022116 30692261 PMC6377499

[B48] Ferrans V. J. (1978). Overview of cardiac pathology in relation to anthracycline cardiotoxicity. Cancer Treat. Rep. 62 (6), 955–961. 352510

[B49] Franke T. F. Kaplan Dr Fau - Cantley L. C. Cantley L. C. (1997). PI3K: downstream AKTion blocks apoptosis. Cell 88 (4), 435–437. 10.1016/s0092-8674(00)81883-8 9038334

[B50] Gabizon A. Martin F. (1997). Polyethylene glycol-coated (pegylated) liposomal doxorubicin. Rationale for use in solid tumours. Drugs 54 (Suppl. 4), 15–21. 10.2165/00003495-199700544-00005 9361957

[B51] Gallo P. Latronico M. V. Gallo P. Grimaldi S. Borgia F. Todaro M. (2008). Inhibition of class I histone deacetylase with an apicidin derivative prevents cardiac hypertrophy and failure. Cardiovasc. Res. 80, 416–424. 10.1093/cvr/cvn215 18697792

[B52] Ganatra S. Nohria A. Shah S. Groarke J. D. Sharma A. Venesy D. (2019). Upfront dexrazoxane for the reduction of anthracycline-induced cardiotoxicity in adults with preexisting cardiomyopathy and cancer: a consecutive case series. Cardiooncology 29 (5), 1. 10.1186/s40959-019-0036-7 32154008 PMC7048095

[B53] Gardai S. J. Hildeman Da Fau - Frankel S. K. Frankel Sk Fau - Whitlock B. B. Frasch S. C. Borregaard N. (2004). Phosphorylation of Bax Ser184 by Akt regulates its activity and apoptosis in neutrophils. J. Biol. Chem. 279 (20), 21085–21095. 10.1074/jbc.M400063200 14766748

[B54] Georgiadis N. Tsarouhas K. Rezaee R. Nepka H. Kass G. E. N. Dorne J. L. C. M. (2020). What is considered cardiotoxicity of anthracyclines in animal studies. Oncol. Rep. 44 (3), 798–818. 10.3892/or.2020.7688 32705236 PMC7388356

[B55] Ghignatti P. Nogueira L. J. Lehnen A. M. Leguisamo N. M. (2021). Cardioprotective effects of exercise training on doxorubicin-induced cardiomyopathy: a systematic review with meta-analysis of preclinical studies. Sci. Rep. 11 (1), 6330. 10.1038/s41598-021-83877-8 33737561 PMC7973566

[B56] Goffart S. von Kleist-Retzow Jc Fau - Wiesner R. J. Wiesner R. J. (2004). Regulation of mitochondrial proliferation in the heart: power-plant failure contributes to cardiac failure in hypertrophy. Cardiovasc. Res. 64 (2), 198–207. 10.1016/j.cardiores.2004.06.030 15485678

[B57] Gong D. Chen X. Fau - Middleditch M. Middleditch M. Fau - Huang L. Reddy S. (2009). Quantitative proteomic profiling identifies new renal targets of copper(II)-selective chelation in the reversal of diabetic nephropathy in rats. Proteomics 9 (18), 4309–4320. 10.1002/pmic.200900285 19634143

[B58] Granger A. Abdullah I. Huebner F. Stout A. Wang T. Huebner T. (2008). Histone deacetylase inhibition reduces myocardial ischemia-reperfusion injury in mice. FASEB J. 22, 3549–3560. 10.1096/fj.08-108548 18606865 PMC2537432

[B59] Granzier H. L. Labeit S. (2004). The giant protein titin: a major player in myocardial mechanics, signaling, and disease. Circulation Res. 94 (3), 284–295. 10.1161/01.RES.0000117769.88862.F8 14976139

[B60] Guo W. A.-O. Liu W. Chen Z. A.-O. Peng S. Shen L. (2017). Tyrosine phosphatase SHP2 negatively regulates NLRP3 inflammasome activation via ANT1-dependent mitochondrial homeostasis. Nat. Commun. 8 (1), 2168. 10.1038/s41467-017-02351-0 29255148 PMC5735095

[B61] Henning Y. A.-O. Blind U. S. Larafa S. Matschke J. Fandrey J. (2022). Hypoxia aggravates ferroptosis in RPE cells by promoting the Fenton reaction. Cell Death Dis. 13 (7), 662. 10.1038/s41419-022-05121-z 35906211 PMC9338085

[B62] Henninger C. Fritz G. (2017). Statins in anthracycline-induced cardiotoxicity: rac and Rho, and the heartbreakers. Cell death Dis. 8, e2564. 10.1038/cddis.2016.418 28102848 PMC5386353

[B63] Henriksen P. A. (2018). Anthracycline cardiotoxicity: an update on mechanisms, monitoring and prevention. Heart 104 (12), 971–977. 10.1136/heartjnl-2017-312103 29217634

[B64] Hertz D. L. Caram M. V. Kidwell K. M. Thibert J. N. Gersch C. Seewald N. J. (2016). Evidence for association of SNPs in ABCB1 and CBR3, but not RAC2, NCF4, SLC28A3 or TOP2B, with chronic cardiotoxicity in a cohort of breast cancer patients treated with anthracyclines. Pharmacogenomics 17, 231–240. 10.2217/pgs.15.162 26799497 PMC5558515

[B65] Hiona A. Lee A. S. Nagendran J. Xie X. Connolly A. J. Robbins R. C. (2011). Pretreatment with angiotensin-converting enzyme inhibitor improves doxorubicin-induced cardiomyopathy via preservation of mitochondrial function. J. Thorac. Cardiovasc. Surg. 142, 396–403. 10.1016/j.jtcvs.2010.07.097 21094500 PMC3173512

[B66] Hoshino A. Mita Y. Fau - Okawa Y. Okawa Y. Fau - Ariyoshi M. Ueyama T. (2013). Cytosolic p53 inhibits Parkin-mediated mitophagy and promotes mitochondrial dysfunction in the mouse heart. Nat. Commun. 4, 2308. 10.1038/ncomms3308 23917356

[B67] Ichikawa Y. Ghanefar M. Bayeva M. Wu R. Khechaduri A. Naga Prasad S. V. (2014). Cardiotoxicity of doxorubicin is mediated through mitochondrial iron accumulation. J. Clin. investigation 124, 617–630. 10.1172/JCI72931 24382354 PMC3904631

[B68] Jang H. S. Noh M. R. Jung E. M. Kim W. Y. Southekal S. Guda C. (2020). Proximal tubule cyclophilin D regulates fatty acid oxidation in cisplatin-induced acute kidney injury. Kidney Int. 97 (2), 327–339. 10.1016/j.kint.2019.08.019 31733829 PMC6983334

[B69] Januchowski R. Wojtowicz K. Andrzejewska M. Zabel M. (2014). Expression of MDR1 and MDR3 gene products in paclitaxel-doxorubicin- and vincristine-resistant cell lines. Biomed. Pharmacother. 68, 111–117. 10.1016/j.biopha.2013.09.004 24140176

[B70] Jiang D. Liang J. Noble P. W. (2011). Hyaluronan as an immune regulator in human diseases. Physiol. Rev. 91, 221–264. 10.1152/physrev.00052.2009 21248167 PMC3051404

[B71] Jourdain A. Martinou J. C. (2009). Mitochondrial outer-membrane permeabilization and remodelling in apoptosis. Int. J. Biochem. Cell Biol. 41 (10), 1884–1889. 10.1016/j.biocel.2009.05.001 19439192

[B72] Kalay N. Basar E. Ozdogru I. Er O. Cetinkaya Y. Dogan A. (2006). Protective effects of carvedilol against anthracycline-induced cardiomyopathy. J. Am. Coll. Cardiol. 48, 2258–2262. 10.1016/j.jacc.2006.07.052 17161256

[B73] Kaya M. G. Ozkan M. Gunebakmaz O. Akkaya H. Kaya E. G. Akpek M. (2013). Protective effects of nebivolol against anthracycline-induced cardiomyopathy: a randomized control study. Int. J. Cardiol. 167, 2306–2310. 10.1016/j.ijcard.2012.06.023 22727976

[B74] Khiati S. Dalla Rosa I. Sourbier C. Ma X. Rao V. A. Neckers L. M. (2014). Mitochondrial topoisomerase I (top1mt) is a novel limiting factor of doxorubicin cardiotoxicity. Clin. Cancer Res. 20 (18), 4873–4881. 10.1158/1078-0432.CCR-13-3373 24714774 PMC4167185

[B75] Khouri M. G. Douglas P. S. Mackey J. R. Martin M. Scott J. M. Scherrer-Crosbie M. (2012). Cancer therapy-induced cardiac toxicity in early breast cancer: addressing the unresolved issues. Circulation 126 (23), 2749–2763. 10.1161/CIRCULATIONAHA.112.100560 23212997 PMC3667651

[B209] Kim Y. Seidman J. G. Seidman C. E. (2022). Genetics of cancer therapy-associated cardiotoxicity. J. Mol. Cell. Cardiol. 167, 85–91. 10.1016/j.yjmcc.2022.03.010 35358500 PMC9107514

[B76] Klumpe I. Savvatis K. Westermann D. Tschöpe C. Rauch U. Landmesser U. (2016). Transgenic overexpression of adenine nucleotide translocase 1 protects ischemic hearts against oxidative stress. J. Mol. Med. Berl. 94 (6), 645–653. 10.1007/s00109-016-1413-4 27080394

[B77] Kong Y. Tannous P. Lu G. Berenji K. Rothermel B. A. Olson E. N. (2006). Suppression of class I and II histone deacetylases blunts pressure-overload cardiac hypertrophy. Circulation 113, 2579–2588. 10.1161/CIRCULATIONAHA.106.625467 16735673 PMC4105979

[B78] Kroemer G. Galluzzi L. Fau - Brenner C. Brenner C. (2007). Mitochondrial membrane permeabilization in cell death. Physiol. Rev. 87 (1), 99–163. 10.1152/physrev.00013.2006 17237344

[B79] Kumarswamy R. Chandna S. (2009). Putative partners in Bax mediated cytochrome-c release: ANT, CypD, VDAC or none of them? Mitochondrion 9 (1), 1–8. 10.1016/j.mito.2008.10.003 18992370

[B80] Lee J. H. Wendorff T. J. Berger J. M. (2017). Resveratrol: a novel type of topoisomerase II inhibitor. J. Biol. Chem. 292 (51), 21011–21022. 10.1074/jbc.M117.810580 29074616 PMC5743075

[B81] Leong S. L. Chaiyakunapruk N. Lee S. W. (2017). Candidate gene association studies of anthracycline-induced cardiotoxicity: a systematic review and meta-analysis. Sci. Rep. 7, 39. 10.1038/s41598-017-00075-1 28232737 PMC5428315

[B82] Li D. L. Wang Z. V. Ding G. Tan W. Luo X. Criollo A. (2016b). Doxorubicin blocks cardiomyocyte autophagic flux by inhibiting lysosome acidification. Circulation 133 (17), 1668–1687. 10.1161/CIRCULATIONAHA.115.017443 26984939 PMC4856587

[B83] Li J. Yan Z. Fau - Fang Q. Fang Q. (2017). A mechanism study underlying the protective effects of cyclosporine-A on lung ischemia-reperfusion injury. Pharmacology 100 (1-2), 83–90. 10.1159/000458760 28501872

[B84] Li K. Sung R. Y. T. Huang W. Z. Yang M. Pong N. H. Lee S. M. (2006). Thrombopoietin protects against *in vitro* and *in vivo* cardiotoxicity induced by doxorubicin. Circulation 113 (18), 2211–2220. 10.1161/CIRCULATIONAHA.105.560250 16651473

[B85] Li L. Hua Y. Ren J. (2012). Short-chain fatty acid propionate alleviates Akt2 knockout-induced myocardial contractile dysfunction. Exp. Diabetes Res. 2012, 851717. 10.1155/2012/851717 21960994 PMC3179899

[B86] Li S. Wang W. Niu T. Wang H. Li B. Shao L. (2014). Nrf2 deficiency exaggerates doxorubicin-induced cardiotoxicity and cardiac dysfunction. Oxid. Med. Cell Longev. 2014, 748524. 10.1155/2014/748524 24895528 PMC4033424

[B87] Li X. Wang H. Yao B. Xu W. Chen J. Zhou X. (2016a). lncRNA H19/miR-675 axis regulates cardiomyocyte apoptosis by targeting VDAC1 in diabetic cardiomyopathy. Sci. Rep. 31 (6), 36340. 10.1038/srep36340 27796346 PMC5087087

[B88] Lien C. Y. Jensen B. T. Hydock D. S. Hayward R. (2015). Short-term exercise training attenuates acute doxorubicin Cardiotoxicity. J. Physiology Biochem. 71, 669–678. 10.1007/s13105-015-0432-x 26403766

[B89] Lim C. C. Zuppinger C. Fau - Guo X. Guo X. Fau - Kuster G. M. Eppenberger H. M. (2004). Anthracyclines induce calpain-dependent titin proteolysis and necrosis in cardiomyocytes. J. Biol. Chem. 279 (9), 8290–8299. 10.1074/jbc.M308033200 14676206

[B90] Linton M. F. Moslehi J. J. Babaev V. R. (2019). Akt signaling in macrophage polarization, survival, and atherosclerosis. Int. J. Mol. Sci. 20 (11), 2703. 10.3390/ijms20112703 31159424 PMC6600269

[B91] Liou G. Y. Storz P. (2010). Reactive oxygen species in cancer. Free Radic. Res. 44 (5), 479–496. 10.3109/10715761003667554 20370557 PMC3880197

[B92] Lipshultz S. E. Giantris A. L. Lipsitz S. R. Dalton V. K. Asselin B. L. Barr R. D. (2002). Doxorubicin administration by continuous infusion is not cardioprotective: the Dana-Farber 91-01 Acute Lymphoblastic Leukemia protocol. J. Clin. Oncol. 20 (6), 1677–1682. 10.1200/JCO.2002.20.6.1677 11896119

[B93] Liu H. Mi Z. Lin L. Wang Y. Zhang Z. Zhang F. (2018). Shifting plant species composition in response to climate change stabilizes grassland primary production. Proc. Natl. Acad. Sci. U. S. A. 115 (16), 4051–4056. 10.1073/pnas.1700299114 29666319 PMC5910805

[B94] Lundy S. D. Zhu W. Z. Regnier M. Laflamme M. A. (2013). Structural and functional maturation of cardiomyocytes derived from human pluripotent stem cells. Stem cells Dev. 22, 1991–2002. 10.1089/scd.2012.0490 23461462 PMC3699903

[B95] Luo M. Guan X. Luczak E. D. Lang D. Kutschke W. Gao Z. (2013). Diabetes increases mortality after myocardial infarction by oxidizing CaMKII. J. Clin. Investigation 123 (3), 1262–1274. 10.1172/JCI65268 23426181 PMC3673230

[B210] Madonna R. (2017). Early diagnosis and prediction of anticancer drug-induced cardiotoxicity: from cardiac imaging to “omics” technologies. Rev. Española Cardiol. 70 (7), 576–582. 10.1016/j.rec.2017.02.001 28246019

[B96] Magdy T. A.-O. X. Jouni M. Kuo H. A.-O. Weddle C. J. Lyra-Leite D. Fonoudi H. (2022). Identification of drug transporter genomic variants and inhibitors that protect against doxorubicin-induced cardiotoxicity. Circulation 145 (4), 279–294. 10.1161/CIRCULATIONAHA.121.055801 34874743 PMC8792344

[B97] Manrique C. R. Park M. Tiwari N. Plana J. C. Garcia M. J. (2017). Diagnostic strategies for early recognition of cancer therapeutics-related cardiac dysfunction. Clin. Med. Insights Cardiol. 11, 1179546817697983. 10.1177/1179546817697983 28469492 PMC5392033

[B98] Marco A. Cassinelli G. Arcamone F. (1981). The discovery of daunorubicin. Cancer Treat. Rep. 65 (Suppl. 4), 3–8. 7049379

[B99] Marques-Aleixo I. Santos-Alves E. Balça M. M. Moreira P. I. Oliveira P. J. Magalhães J. (2016). Physical exercise mitigates doxorubicin-induced brain cortex and cerebellum mitochondrial alterations and cellular quality control signaling. Mitochondrion 26, 43–57. 10.1016/j.mito.2015.12.002 26678157

[B100] Marques-Aleixo I. Santos-Alves E. Mariani D. Rizo-Roca D. Padrão A. I. Rocha-Rodrigues S. (2015). Physical exercise prior and during treatment reduces sub-chronic doxorubicin-induced mitochondrial toxicity and oxidative stress. Mitochondrion 20, 22–33. 10.1016/j.mito.2014.10.008 25446396

[B101] Marty M. Espié M. Fau - Llombart A. Llombart A. Fau - Monnier A. Stahalova V. (2006). Multicenter randomized phase III study of the cardioprotective effect of dexrazoxane (Cardioxane) in advanced/metastatic breast cancer patients treated with anthracycline-based chemotherapy. Ann. Oncol. 17 (4), 614–622. 10.1093/annonc/mdj134 16423847

[B102] Masters S. C. Yang H. Datta S. R. Greenberg M. E. Fu H. (2004). 14-3-3 inhibits Bad-induced cell death through interaction with serine-136. Mol. Pharmacol. 60 (6), 1325–31. 10.1124/mol.60.6.1325 11723239

[B103] Meng L. Lin H. Zhang J. Sun Z. Gao F. (2019). Doxorubicin induces cardiomyocyte pyroptosis via the TINCR-mediated posttranscriptional stabilization of NLR family pyrin domain containing 3. J. Mol. Cell Cardiol. 136, 15–26. 10.1016/j.yjmcc.2019.08.009 31445005

[B104] Menon A. V. Kim J. (2022). Iron promotes cardiac doxorubicin retention and toxicity through downregulation of the mitochondrial exporter ABCB8. Front. Pharmacol. 11. 10.3389/fphar.2022.817951 35359834 PMC8963208

[B105] Michel L. Totzeck M. Rassaf T. (2022). ESC-Leitlinie 2022 onkologische Kardiologie. Herz 48, 15–22. 10.1007/s00059-022-05149-z 36441175

[B106] Middleman E. Luce J. Frei E. (1971). Clinical trials with adriamycin. Cancer 28, 844–850. 10.1002/1097-0142(1971)28:4<844::aid-cncr2820280407>3.0.co;2-9 5111740

[B107] Mielke C. Lefort N. Fau - McLean C. G. McLean Cg Fau - Cordova J. M. Langlais P. R. Bordner A. J. (2014). Adenine nucleotide translocase is acetylated *in vivo* in human muscle: modeling predicts a decreased ADP affinity and altered control of oxidative phosphorylation. Biochemistry 53 (23), 3817–3829. 10.1021/bi401651e 24884163 PMC4067143

[B108] Milano G. Biemmi V. Lazzarini E. Balbi C. Ciullo A. Bolis S. (2020). Intravenous administration of cardiac progenitor cell-derived exosomes protects against doxorubicin/trastuzumab-induced cardiac toxicity. Cardiovasc. Res. 116 (2), 383–392. 10.1093/cvr/cvz108 31098627

[B109] Minotti G. Menna P. Fau - Salvatorelli E. Salvatorelli E. Fau - Cairo G. (2004). Anthracyclines: molecular advances and pharmacologic developments in antitumor activity and cardiotoxicity. Pharmacol. Rev. 56 (2), 185–229. 10.1124/pr.56.2.6 15169927

[B110] Minotti G. Recalcati S. Fau - Mordente A. Mordente A. Fau - Liberi G. Mancuso C. (1998). The secondary alcohol metabolite of doxorubicin irreversibly inactivates aconitase/iron regulatory protein-1 in cytosolic fractions from human myocardium. Faseb J. 12 (7), 541–552. 10.1096/fasebj.12.7.541 9576481

[B111] Mira M. L. Silva M. M. Manso C. F. (1994). The scavenging of oxygen free radicals by angiotensin converting enzyme inhibitors: the importance of the sulfhydryl group in the chemical structure of the compounds. Ann. N. Y. Acad. Sci. 723, 439–441. 10.1111/j.1749-6632.1994.tb36771.x 8030906

[B112] Miranda C. J. Makui H. Fau - Soares R. J. Soares R. J. Mui J. Vali H. (2003). Hfe deficiency increases susceptibility to cardiotoxicity and exacerbates changes in iron metabolism induced by doxorubicin. Blood 102 (7), 2574–2580. 10.1182/blood-2003-03-0869 12805055

[B113] Misaka T. Yoshihisa A. Takeishi Y. (2019). Titin in muscular dystrophy and cardiomyopathy: urinary titin as a novel marker. Clin. Chim. Acta 495 (2019), 123–128. 10.1016/j.cca.2019.04.005 30959043

[B114] Moyal L. Goldfeiz N. Gorovitz B. Rephaeli A. Tarasenko N. (2018). AN-7, a butyric acid prodrug, sensitizes cutaneous T-cell lymphoma cell lines to doxorubicin via inhibition of DNA double strand breaks repair. Investig. New Drugs 36, 1–9. 10.1007/s10637-017-0500-x 28884410

[B115] Mulrooney D. A. Yeazel M. W. Kawashima T. Mertens A. C. Mitby P. Stovall M. (2009). Cardiac outcomes in a cohort of adult survivors of childhood and adolescent cancer: retrospective analysis of the Childhood Cancer Survivor Study cohort. BMJ Clin. Res. 339, b4606. 10.1136/bmj.b4606 19996459 PMC3266843

[B116] Myers C. E. Gianni L. Simone C. B. Klecker R. Greene R. (1982). Oxidative destruction of erythrocyte ghost membranes catalyzed by the doxorubicin-iron complex. Biochemistry 21 (8), 1707–1712. 10.1021/bi00537a001 6805506

[B117] Naaktgeboren W. A.-O. Binyam D. Stuiver M. A.-O. Aaronson N. K. Teske A. J. van Harten W. H. (2021). Efficacy of physical exercise to offset anthracycline-induced cardiotoxicity: a systematic review and meta-analysis of clinical and preclinical studies. J. Am. Heart Assoc. 10 (17), e021580. 10.1161/JAHA.121.021580 34472371 PMC8649276

[B118] Nabati M. Janbabai G. Baghyari S. Esmaili K. Yazdani J. (2017). Cardioprotective effects of carvedilol in inhibiting doxorubicin-induced cardiotoxicity. J. Cardiovasc. Pharmacol. 69, 279–285. 10.1097/FJC.0000000000000470 28141699

[B119] Nabati M. Janbabai G. Esmailian J. Yazdani J. (2019). Effect of rosuvastatin in preventing chemotherapy-induced cardiotoxicity in women with breast cancer: a randomized, single-blind, placebo-controlled trial. J. Cardiovasc. Pharmacol. Ther. 24, 233–241. 10.1177/1074248418821721 30599756

[B120] Nebigil C. G. Désaubry L. (2018). Updates in anthracycline-mediated cardiotoxicity. Front. Pharmacol. 12 (9), 1262. 10.3389/fphar.2018.01262 30483123 PMC6240592

[B121] Nechushtan A. Smith C. L. Hsu Y. T. Youle R. J. (1999). Conformation of the Bax C-terminus regulates subcellular location and cell death. Embo J. 18 (9), 2330–2341. 10.1093/emboj/18.9.2330 10228148 PMC1171316

[B122] Nunes-Nesi A. Araújo Wl Fau - Obata T. Obata T. Fau - Fernie A. R. Fernie A. R. (2013). Regulation of the mitochondrial tricarboxylic acid cycle. Curr. Opin. Plant Biol. 16 (3), 335–343. 10.1016/j.pbi.2013.01.004 23462640

[B123] Oh C. A.-O. Cho S. A.-O. Jang J. A.-O. Kim H. Chun S. Choi M. (2019). Cardioprotective potential of an SGLT2 inhibitor against doxorubicin-induced heart failure. Korean Circ. J. 49 (12), 1183–1195. 10.4070/kcj.2019.0180 31456369 PMC6875592

[B124] Oh J. Lee B. S. Lim G. Lim H. Lee C. J. Park S. (2020). Atorvastatin protects cardiomyocyte from doxorubicin toxicity by modulating survivin expression through FOXO1 inhibition. J. Mol. Cell. Cardiol. 138, 244–255. 10.1016/j.yjmcc.2019.12.007 31866378

[B125] Orsi B. D' Mateyka J. Prehn J. H. M. (2017). Control of mitochondrial physiology and cell death by the Bcl-2 family proteins Bax and Bok. Neurochem. Int. 109, 162–170. 10.1016/j.neuint.2017.03.010 28315370

[B126] Palmieri F. (2013). The mitochondrial transporter family SLC25: identification, properties and physiopathology. Mol. Asp. Med. 34 (2), 465–484. 10.1016/j.mam.2012.05.005 23266187

[B127] Parker M. A. King V. Fau - Howard K. P. Howard K. P. (2001). Nuclear magnetic resonance study of doxorubicin binding to cardiolipin containing magnetically oriented phospholipid bilayers. Biochim. Biophys. Acta 1514 (2), 206–216. 10.1016/s0005-2736(01)00371-6 11557021

[B128] Peoples J. N. Saraf A. Ghazal N. Pham T. T. Kwong J. Q. (2019). Mitochondrial dysfunction and oxidative stress in heart disease. Exp. Mol. Med. 51 (12), 1–13. 10.1038/s12276-019-0355-7 31857574 PMC6923355

[B129] Plana J. C. Galderisi M. Barac A. Ewer M. S. Ky B. Scherrer-Crosbie M. (2014). Expert consensus for multimodality imaging evaluation of adult patients during and after cancer therapy: a report from the American Society of Echocardiography and the European Association of Cardiovascular Imaging. J. Am. Soc. Echocardiogr. 27 (9), 911–939. 10.1016/j.echo.2014.07.012 25172399

[B130] Reichardt P. Tabone M. D. Mora J. Morland B. Jones R. L. (2018). Risk-benefit of dexrazoxane for preventing anthracycline-related cardiotoxicity: re-evaluating the European labeling. Future Oncol. 14, 2663–2676. 10.2217/fon-2018-0210 29747541

[B131] Rephaeli A. Waks-Yona S. Nudelman A. Tarasenko I. Tarasenko N. Phillips D. R. (2007). Anticancer prodrugs of butyric acid and formaldehyde protect against doxorubicin-induced cardiotoxicity. Br. J. Cancer 96, 1667–1674. 10.1038/sj.bjc.6603781 17473824 PMC2359917

[B132] Russo M. Della Sala A. Tocchetti C. G. Porporato P. E. Ghigo A. (2021). Metabolic aspects of anthracycline cardiotoxicity. Curr. Treat. Options Oncol. 22 (2), 18. 10.1007/s11864-020-00812-1 33547494 PMC7864817

[B133] Russo M. Guida F. Paparo L. Trinchese G. Aitoro R. Avagliano C. (2019). The novel butyrate derivative phenylalanine-butyramide protects from doxorubicin-induced cardiotoxicity. Eur. J. heart Fail. 21 (4), 519–528. 10.1002/ejhf.1439 30843309

[B134] Sabatino J. De Rosa S. A.-O. X. Tammè L. Iaconetti C. Sorrentino S. Polimeni A. (2020). Empagliflozin prevents doxorubicin-induced myocardial dysfunction. Cardiovasc Diabetol. 19 (1), 66. 10.1186/s12933-020-01040-5 32414364 PMC7229599

[B135] Sala V. Della Sala A. Hirsch E. Ghigo A. (2020). Signaling pathways underlying anthracycline cardiotoxicity. Antioxid. Redox Signal 32 (15), 1098–1114. 10.1089/ars.2020.8019 31989842

[B136] Saleem T. S. Bharani K. Gauthaman K. (2010). ACE inhibitors - angiotensin II receptor antagonists: a useful combination therapy for ischemic heart disease. Open access Emerg. Med. 2, 51–59. 10.2147/oaem.s10507 27147838 PMC4806827

[B137] Sarvazyan N. (1996). Visualization of doxorubicin-induced oxidative stress in isolated cardiac myocytes. Am. J. Physiol. 271 (5), 2079–2085. 10.1152/ajpheart.1996.271.5.H2079 8945928

[B138] Sawicki G. Leon H. Fau - Sawicka J. Sawicka J. F.-S. M. Schulze C. J. Scott P. G. (2005). Degradation of myosin light chain in isolated rat hearts subjected to ischemia-reperfusion injury: a new intracellular target for matrix metalloproteinase-2. Circulation 112 (4), 544–552. 10.1161/CIRCULATIONAHA.104.531616 16027249

[B139] Sawicki K. T. Sala V. Prever L. Hirsch E. Ardehali H. Ghigo A. (2021). Preventing and treating anthracycline cardiotoxicity: new insights. Annu. Rev. Pharmacol. Toxicol. 6 (61), 309–332. 10.1146/annurev-pharmtox-030620-104842 33022184

[B140] Sawyer D. B. Zuppinger C. Fau - Miller T. A. Miller T. A. Suter T. M. (2002). Modulation of anthracycline-induced myofibrillar disarray in rat ventricular myocytes by neuregulin-1beta and anti-erbB2: potential mechanism for trastuzumab-induced cardiotoxicity. Circulation 105 (13), 1551–1554. 10.1161/01.cir.0000013839.41224.1c 11927521

[B141] Schachter M. (2005). Chemical, pharmacokinetic and pharmacodynamic properties of statins: an update. Fundam. Clin. Pharmacol. 19, 117–125. 10.1111/j.1472-8206.2004.00299.x 15660968

[B142] Schinzel A. C. Takeuchi O. Huang Z. Fisher J. K. Zhou Z. Rubens J. (2005). Cyclophilin D is a component of mitochondrial permeability transition and mediates neuronal cell death after focal cerebral ischemia. Proc. Natl. Acad. Sci. U. S. A. 102 (34), 12005–10. 10.1073/pnas.0505294102 16103352 PMC1189333

[B211] Schirone L. D’Ambrosio L. Forte M. Genovese R. Schiavon S. Spinosa G. (2022). Mitochondria and doxorubicin-induced cardiomyopathy: a complex interplay. Cells 11 (13), 2000. 10.3390/cells11132000 35805084 PMC9266202

[B143] Schmieder R. E. (2005). Mechanisms for the clinical benefits of angiotensin II receptor blockers. Am. J. Hypertens. 18, 720–730. 10.1016/j.amjhyper.2004.11.032 15882557

[B144] Schwebe M. Ameling S. Hammer E. Monzel J. V. Bonitz K. Budde S. (2015). Protective effects of endothelin receptor A and B inhibitors against doxorubicin-induced cardiomyopathy. Biochem. Pharmacol. 94 (2), 109–129. 10.1016/j.bcp.2015.01.014 25660617

[B145] Seidman A. Hudis C. Fau - Pierri M. K. Pierri Mk F.-S. S. Paton V. Ashby M. (2002). Cardiac dysfunction in the trastuzumab clinical trials experience. J. Clin. Oncol. 20 (5), 1215–1221. 10.1200/JCO.2002.20.5.1215 11870163

[B146] Shan K. Lincoff Am Fau - Young J. B. Young J. B. (1996). Anthracycline-induced cardiotoxicity. Ann. Intern Med. 125 (1), 47–58. 10.7326/0003-4819-125-1-199607010-00008 8644988

[B147] Shirinbayan V. Roshan V. D. (2012). Pretreatment effect of running exercise on HSP70 and DOX-induced cardiotoxicity. Asian Pac. J. Cancer Prev. 13, 5849–5855. 10.7314/apjcp.2012.13.11.5849 23317268

[B148] Shoshan-Barmatz V. Ben-Hail D. Admoni L. Krelin Y. Tripathi S. S. (2015). The mitochondrial voltage-dependent anion channel 1 in tumor cells. Biochim. Biophys. Acta 1848 (10), 2547–2575. 10.1016/j.bbamem.2014.10.040 25448878

[B149] Shoshan-Barmatz V. De Pinto V. Fau - Zweckstetter M. Zweckstetter M. Fau - Raviv Z. Arbel N. (2010). VDAC, a multi-functional mitochondrial protein regulating cell life and death. Mol. Asp. Med. 31 (3), 227–285. 10.1016/j.mam.2010.03.002 20346371

[B150] Silber J. H. (2004). Can dexrazoxane reduce myocardial injury in anthracycline-treated children with acute lymphoblastic leukemia? Nat. Clin. Pract. Oncol. 1 (1), 16–17. 10.1038/ncponc0023 16264791

[B151] Skommer J. Wlodkowic D. Fau - Deptala A. Deptala A. (2007). Larger than life: mitochondria and the bcl-2 family. Leuk. Res. 31 (3), 277–286. 10.1016/j.leukres.2006.06.027 16911824

[B152] Soengas M. S. Alarcón Rm Fau - Yoshida H. Yoshida H. Fau - Giaccia A. J. Mak T. W. (1999). Apaf-1 and caspase-9 in p53-dependent apoptosis and tumor inhibition. Science 284 (5411), 156–159. 10.1126/science.284.5411.156 10102818

[B153] Songbo M. Lang H. Xinyong C. Bin X. Ping Z. Liang S. (2019). Oxidative stress injury in doxorubicin-induced cardiotoxicity. Toxicol. Lett. 307, 41–48. 10.1016/j.toxlet.2019.02.013 30817977

[B154] Spallarossa P. Garibaldi S. Altieri P. Fabbi P. Manca V. Nasti S. (2004). Carvedilol prevents doxorubicin-induced free radical release and apoptosis in cardiomyocytes *in vitro* . J. Mol. Cell. Cardiol. 37, 837–846. 10.1016/j.yjmcc.2004.05.024 15380674

[B155] Stanley W. C. Recchia F. A. Lopaschuk G. D. (2005). Myocardial substrate metabolism in the normal and failing heart. Physiol. Rev. 85, 1093–1129. 10.1152/physrev.00006.2004 15987803

[B156] Stěrba M. Popelová O. Fau - Vávrová A. Vávrová A. Fau - Jirkovský E. Geršl V. (2013). Oxidative stress, redox signaling, and metal chelation in anthracycline cardiotoxicity and pharmacological cardioprotection. Antioxid. Redox Signal 18 (8), 899–929. 10.1089/ars.2012.4795 22794198 PMC3557437

[B157] Storr S. J. Carragher No Fau - Frame M. C. Frame Mc Fau - Parr T. Martin S. G. (2011). The calpain system and cancer. Nat. Rev. Cancer 11 (5), 364–374. 10.1038/nrc3050 21508973

[B158] Su É. A.-O. Villard C. Manneville J. A.-O. (2023). Mitochondria: at the crossroads between mechanobiology and cell metabolism. Biol. Cell 115 (9), e2300010. 10.1111/boc.202300010 37326132

[B159] Sultan S. Murarka S. Jahangir A. Mookadam F. Tajik A. J. (2017). Chelation therapy in cardiovascular disease: an update. Expert Rev. Clin. Pharmacol. 10 (8), 843–854. 10.1080/17512433.2017.1339601 28597699

[B160] Sung M. M. Schulz Cg Fau - Wang W. Wang W. Fau - Sawicki G. Bautista-López N. L. Schulz R. (2007). Matrix metalloproteinase-2 degrades the cytoskeletal protein alpha-actinin in peroxynitrite mediated myocardial injury. J. Mol. Cell Cardiol. 43 (4), 429–436. 10.1016/j.yjmcc.2007.07.055 17854826

[B161] Swain S. M. Vici P. (2004). The current and future role of dexrazoxane as a cardioprotectant in anthracycline treatment: expert panel review. J. cancer Res. Clin. Oncol. 130, 1–7. 10.1007/s00432-003-0498-7 14564513 PMC12161775

[B162] Swain S. M. Whaley F. S. Gerber M. C. Weisberg S. York M. Spicer D. (1997). Cardioprotection with dexrazoxane for doxorubicin-containing therapy in advanced breast cancer. J. Clin. Oncol. 15, 1318–1332. 10.1200/JCO.1997.15.4.1318 9193323

[B163] Swain S. M. Whaley F. S. Ewer M. S. (2003). Congestive heart failure in patients treated with doxorubicin: a retrospective analysis of three trials. Cancer 97 (11), 2869–2879. 10.1002/cncr.11407 12767102

[B164] Tan C. Tasaka H. Tasaka H. Yu K. P. Yu F. Murphy M. L. (1967). Daunomycin, an antitumor antibiotic, in the treatment of neoplastic disease. Clinical evaluation with special reference to childhood leukemia. Cancer 20 (3), 333–353. 10.1002/1097-0142(1967)20:3<333::aid-cncr2820200302>3.0.co;2-k 4290058

[B165] Tanihata J. Nishioka N. Inoue T. Bando K. Minamisawa S. (2019). Urinary titin is increased in patients after cardiac surgery. Front. Cardiovasc. Med. 8 (6), 7. 10.3389/fcvm.2019.00007 30800662 PMC6375839

[B166] Tarasenko N. Kessler-Icekson G. Boer P. Inbal A. Schlesinger H. Phillips D. R. (2012). The histone deacetylase inhibitor butyroyloxymethyl diethylphosphate (AN-7) protects normal cells against toxicity of anticancer agents while augmenting their anticancer activity. Investig. New Drugs 30, 130–143. 10.1007/s10637-010-9542-z 20862515

[B167] Telli M. L. Hunt S. A. Carlson R. W. Guardino A. E. (2007). Trastuzumab-related cardiotoxicity: calling into question the concept of reversibility. J. Clin. Oncol. 25 (23), 3525–3533. 10.1200/JCO.2007.11.0106 17687157

[B168] Tewey K. M. Rowe T. C. Yang L. Halligan B. D. Liu L. F. (1984). Adriamycin-induced DNA damage mediated by mammalian DNA topoisomerase II. Science 226 (4673), 466–468. 10.1126/science.6093249 6093249

[B169] Tremaroli V. Bäckhed F. (2012). Functional interactions between the gut microbiota and host metabolism. Nature 489, 242–249. 10.1038/nature11552 22972297

[B170] Uko N. E. Güner O. F. Matesic D. F. Bowen J. P. (2020). Akt pathway inhibitors. Curr. Top. Med. Chem. 20 (10), 883–900. 10.2174/1568026620666200224101808 32091335

[B171] van Dalen E. C. Caron H. N. Dickinson H. O. Kremer L. C. (2011). Cardioprotective interventions for cancer patients receiving anthracyclines. Cochrane Database Syst. Rev. 2011 (6), CD003917. 10.1002/14651858.CD003917.pub3 18425895

[B172] Varga Z. V. Ferdinandy P. Liaudet L. Pacher P. (2015). Drug-induced mitochondrial dysfunction and cardiotoxicity. Am. J. Physiol. Heart Circ. Physiol. 309 (9), 1453–1467. 10.1152/ajpheart.00554.2015 26386112 PMC4666974

[B173] Vavrova A. Jansova H. Fau - Mackova E. Mackova E. Fau - Machacek M. Tichotova L. (2013). Catalytic inhibitors of topoisomerase II differently modulate the toxicity of anthracyclines in cardiac and cancer cells. PLoS One 8 (10), e76676. 10.1371/journal.pone.0076676 24116135 PMC3792022

[B174] Vega R. B. Horton J. L. Kelly D. P. (2015). Maintaining ancient organelles: mitochondrial biogenesis and maturation. Circ. Res. 116 (11), 1820–1834. 10.1161/CIRCRESAHA.116.305420 25999422 PMC4443496

[B175] Venturini M. Michelotti A. Fau - Del Mastro L. Del Mastro L. Fau - Gallo L. Garrone O. (1996). Multicenter randomized controlled clinical trial to evaluate cardioprotection of dexrazoxane versus no cardioprotection in women receiving epirubicin chemotherapy for advanced breast cancer. J. Clin. Oncol. 14 (12), 3112–3120. 10.1200/JCO.1996.14.12.3112 8955656

[B176] Visscher H. Ross C. J. Rassekh S. R. Barhdadi A. Dubé M. P. Al-Saloos H. (2012). Pharmacogenomic prediction of anthracycline-induced cardiotoxicity in children. J. Clin. Oncol. 30, 1422–1428. 10.1200/JCO.2010.34.3467 21900104

[B177] Von Hoff D. D. Layard M. W. Basa P. Basa P. Fau - Davis H. L. Rozencweig M. (1979). Risk factors for doxorubicin-induced congestive heart failure. Ann. Intern Med. 91 (5), 710–717. 10.7326/0003-4819-91-5-710 496103

[B178] Vulsteke C. Pfeil A. M. Maggen C. Schwenkglenks M. Pettengell R. Szucs T. D. (2015). Clinical and genetic risk factors for epirubicin-induced cardiac toxicity in early breast cancer patients. Breast cancer Res. Treat. 152, 67–76. 10.1007/s10549-015-3437-9 26017071

[B179] Wang F. A.-O. Chandra J. A.-O. Kleinerman E. S. (2021). Exercise intervention decreases acute and late doxorubicin-induced cardiotoxicity. Cancer Med. 10 (21), 7572–7584. 10.1002/cam4.4283 34523825 PMC8559466

[B180] Wang J. C. (2002). Cellular roles of DNA topoisomerases: a molecular perspective. Nat. Rev. Mol. Cell Biol. 3 (6), 430–440. 10.1038/nrm831 12042765

[B181] Wang W. Schulze C. J. Suarez-Pinzon W. L. Suarez-Pinzon W. L. Sawicki G. Schulz R. (2002). Intracellular action of matrix metalloproteinase-2 accounts for acute myocardial ischemia and reperfusion injury. Circulation 106 (12), 1543–1549. 10.1161/01.cir.0000028818.33488.7b 12234962

[B182] Wang X. Liu W. Sun C. L. Armenian S. H. Hakonarson H. Hageman L. (2014). Hyaluronan synthase 3 variant and anthracycline-related cardiomyopathy: a report from the children's oncology group. J. Clin. Oncol. 32, 647–653. 10.1200/JCO.2013.50.3557 24470002 PMC3927733

[B183] Wang Y. D. Zhang Y. Sun B. Leng X. W. Li Y. J. Ren L. Q. (2018). Cardioprotective effects of rutin in rats exposed to pirarubicin toxicity. J. Asian Nat. Prod. Res. 20 (4), 361–373. 10.1080/10286020.2017.1394292 29078725

[B184] Winterbourn C. C. (1995). Toxicity of iron and hydrogen peroxide: the Fenton reaction. Toxicol. Lett. 82-83, 969–974. 10.1016/0378-4274(95)03532-x 8597169

[B185] Wojnowski L. Kulle B. Schirmer M. Schlüter G. Schmidt A. Rosenberger A. (2005). NAD(P)H oxidase and multidrug resistance protein genetic polymorphisms are associated with doxorubicin-induced cardiotoxicity. Circulation 112, 3754–3762. 10.1161/CIRCULATIONAHA.105.576850 16330681

[B186] Wonders K. Y. Hydock D. S. Schneider C. M. Hayward R. (2008). Acute exercise protects against doxorubicin cardiotoxicity. Integr. Cancer Ther. 7, 147–154. 10.1177/1534735408322848 18815146

[B187] Xin M. Deng X. (2005). Nicotine inactivation of the proapoptotic function of Bax through phosphorylation. J. Biol. Chem. 280 (11), 10781–10789. 10.1074/jbc.M500084200 15642728

[B188] Xu H. Guan N. A.-O. Ren Y. L. Wei Q. J. Tao Y. H. Yang G. S. (2018). IP3R-Grp75-VDAC1-MCU calcium regulation axis antagonists protect podocytes from apoptosis and decrease proteinuria in an Adriamycin nephropathy rat model. BMC Nephrol. 19 (1), 140–151. 10.1186/s12882-018-0940-3 29907098 PMC6003198

[B189] Xu X. Persson Hl Fau - Richardson D. R. Richardson D. R. (2005). Molecular pharmacology of the interaction of anthracyclines with iron. Mol. Pharmacol. 68 (2), 261–271. 10.1124/mol.105.013383 15883202

[B190] Yamaguchi H. Wang H. G. (2001). The protein kinase PKB/Akt regulates cell survival and apoptosis by inhibiting Bax conformational change. Oncogene 20 (53), 7779–7786. 10.1038/sj.onc.1204984 11753656

[B191] Yang M. Fu J. D. Zou J. Sridharan D. Zhao M. T. Singh H. (2022). Assessment of mitophagy in human iPSC-derived cardiomyocytes. Autophagy 18 (10), 2481–2494. 10.1080/15548627.2022.2037920 35220905 PMC9542630

[B192] Yeh E. T. Bickford C. L. (2009). Cardiovascular complications of cancer therapy: incidence, pathogenesis, diagnosis, and management. J. Am. Coll. Cardiol. 53 (24), 2231–2247. 10.1016/j.jacc.2009.02.050 19520246

[B193] Yin J. Guo J. Zhang Q. Cui L. Zhang L. Zhang T. (2018). Doxorubicin-induced mitophagy and mitochondrial damage is associated with dysregulation of the PINK1/parkin pathway. Toxicol Vitro 51, 1–10. 10.1016/j.tiv.2018.05.001 29729358

[B194] Yu X. Ruan Y. Huang X. Dou L. Lan M. Cui J. (2020). Dexrazoxane ameliorates doxorubicin-induced cardiotoxicity by inhibiting both apoptosis and necroptosis in cardiomyocytes. Biochem. Biophys. Res. Commun. 523 (1), 140–146. 10.1016/j.bbrc.2019.12.027 31837803

[B195] Zaib S. Hayyat A. Ali N. Gul A. Naveed M. Khan I. (2022). Role of mitochondrial membrane potential and lactate dehydrogenase A in apoptosis. Anticancer Agents Med. Chem. 22 (11), 2048–2062. 10.2174/1871520621666211126090906 34825878

[B196] Zhang Q. L. Yang J. J. Zhang H. S. (2019). Carvedilol (CAR) combined with carnosic acid (CAA) attenuates doxorubicin-induced cardiotoxicity by suppressing excessive oxidative stress, inflammation, apoptosis and autophagy. Biomed. Pharmacother. 109, 71–83. 10.1016/j.biopha.2018.07.037 30396094

[B197] Zhang S. Liu X. Bawa-Khalfe T. Bawa-Khalfe T. Lu L.-S. Liu L. F. (2012). Identification of the molecular basis of doxorubicin-induced cardiotoxicity. Nat. Med. 18 (11), 1639–1642. 10.1038/nm.2919 23104132

[B198] Zhang T. Zhang Y. Cui M. A.-O. Jin L. Wang Y. Lv F. (2016a). CaMKII is a RIP3 substrate mediating ischemia- and oxidative stress-induced myocardial necroptosis. Nat. Med. 22 (2), 175–182. 10.1038/nm.4017 26726877

[B199] Zhang X. Hu C. Kong C. Y. Song P. Wu H. M. Xu S. C. (2020b). FNDC5 alleviates oxidative stress and cardiomyocyte apoptosis in doxorubicin-induced cardiotoxicity via activating AKT. Cell Death Differ. 27 (2), 540–555. 10.1038/s41418-019-0372-z 31209361 PMC7206111

[B200] Zhang Y. Ma C. Liu C. Wei F. (2020a). Luteolin attenuates doxorubicin-induced cardiotoxicity by modulating the PHLPP1/AKT/Bcl-2 signalling pathway. PeerJ 8, e8845. 10.7717/peerj.8845 32435528 PMC7224230

[B201] Zhang Y. Yu Z. Jiang D. Liang X. Liao S. Zhang Z. (2016b). iPSC-MSCs with high intrinsic MIRO1 and sensitivity to TNF-α yield efficacious mitochondrial transfer to rescue anthracycline-induced cardiomyopathy. Stem Cell Rep. 7 (4), 749–763. 10.1016/j.stemcr.2016.08.009 27641650 PMC5063626

[B202] Zhao L. Qi Y. Xu L. Tao X. Han X. Yin L. (2018). MicroRNA-140-5p aggravates doxorubicin-induced cardiotoxicity by promoting myocardial oxidative stress via targeting Nrf2 and Sirt2. Redox Biol. 15, 284–296. 10.1016/j.redox.2017.12.013 29304479 PMC5975069

[B203] Zhu W. Soonpaa Mh Fau - Chen H. Chen H. Fau - Shen W. Liechty E. A. (2009). Acute doxorubicin cardiotoxicity is associated with p53-induced inhibition of the mammalian target of rapamycin pathway. Circulation 119 (1), 99–106. 10.1161/CIRCULATIONAHA.108.799700 19103993 PMC2630181

[B204] Zhuang J. Ma W. Lago C. U. Hwang P. M. (2012). Metabolic regulation of oxygen and redox homeostasis by p53: lessons from evolutionary biology? Free Radic. Biol. Med. 53 (6), 1279–1285. 10.1016/j.freeradbiomed.2012.07.026 22841759 PMC3444283

[B205] Zilinyi R. Czompa A. Czegledi A. Gajtko A. Pituk D. Lekli I. (2018). The cardioprotective effect of metformin in doxorubicin-induced cardiotoxicity: the role of autophagy. Molecules 23, 1184. 10.3390/molecules23051184 29762537 PMC6100061

