# Correction: An update of the molecular mechanisms underlying anthracycline induced cardiotoxicity

**DOI:** 10.3389/fphar.2025.1708198

**Published:** 2025-09-30

**Authors:** Sicong Xie, Yuwei Sun, Xuan Zhao, Yiqun Xiao, Fei Zhou, Liang Lin, Wei Wang, Bin Lin, Zun Wang, Zixuan Fang, Lei Wang, Yang Zhang

**Affiliations:** ^1^ Department of Rehabilitation Medicine, School of Acupuncture-Moxibustion and Tuina and School of Health Preservation and Rehabilitation, Nanjing University of Chinese Medicine, Nanjing, China; ^2^ Department of General Surgery, Jiangsu Province Hospital of Chinese Medicine, Affiliated Hospital of Nanjing University of Chinese Medicine, Nanjing, China; ^3^ College of Electronic and Optical Engineering and College of Flexible Electronics, Future Technology, Nanjing University of Posts and Telecommunications, Nanjing, China; ^4^ Key Laboratory of Intelligent Pharmacy and Individualized Therapy of Huzhou, Department of Pharmacy, Changxing People’s Hospital, Huzhou, China

**Keywords:** anthracycline, cardiotoxicity, mitochondria, DNA, signal pathway

There was a mistake in the captions of **Figures 1–7** as published. The figure sources were not credited in the respective captions and permission information was omitted. The corrected captions of Figures 1–7 appear below.

FIGURE 1 Stages in the course of Anthracycline-induced ventricular dysfunction. (1) Primary prevention is possible at this stage by reducing risk factors in high-risk populations (such as those receiving anticancer therapy). (2) Secondary prevention is possible at this stage to reduce the effects of the treatment-induced injury. (3) Secondary prevention is also possible at this stage. (4) Clinically significant conduction and rhythm abnormalities might be observed. (5) Radical therapies might be required at this stage (such as heart transplant) if there is failure of medical management. Preventive strategies are progressively less effective as the toxicity increases. Treatment strategies have a greater impact when used to treat themore-diseased heart, but have longer effects if initiated early. Reproduced with permission from Madonna (2017), Copyright 2017 Sociedad Española de Cardiología. Published by Elsevier España S.L.U. All rights reserved.

FIGURE 2 Mechanisms of action of doxorubicin (DOX). DOX intercalates between strands of DNA double helix. The formation of a ternary complex (TOP2-DOX-DNA) prevents enzyme turnover. The latter blocks the catalytic cycle after DNA is cleaved and before DNA relegation. DOX biotransformation results in the formation of ROS. Reproduced with permission from Corremans et al. (2019), Copyright 2018 John Wiley and Sons Australia, Ltd.

FIGURE 3 Doxorubicin (DOX) undergoes redoxcycling catalysed by NADPH-Cytochrome P-450 reductase. **(A)** The One-electron (1e-) reduction of the quinone compound leads to redox cycling and generation of superoxide anion radical. **(B)** O2-undergoes dismutation to form H2O2 either spontaneously or catalysed by superoxide dismutase. **(C)** H2O2 then reacts with the transition metal ion Fe2+, giving rise to -OH. Free Fe2+ is toxic to cells as catalyst in the formation of free radicals from ROS via the Fenton reaction. Reproduced with permission from Corremans et al. (2019), Copyright 2018 John Wiley and Sons Australia, Ltd.

FIGURE 4 Anthracycline drugs induced changes in mitochondrial membrane permeability and induced cell death. **(A)** Mitochondrial permeability transition pore (MPTP) structure. It was recently proposed that the MPTP is generally considered to be a complex channel composed of several proteins, including voltage-dependent anion channel 1 (VDAC1) in the outer membrane, adenine nucleotide translocase 1 (ANT1) in the inner membrane, and cyclophilin D (CYPD) in the mitochondrial matrix. In addition, the MPTP can be regulated by other components, such as hexokinase (HK), creatine kinase (CK), and peripheral-type benzodiazepine receptors (PBR). Both antiapoptotic and proapoptotic members of the Bcl-2 family modulate the activity of MPTP (antiapoptotic members of the Bcl-2 family, including Bcl-2 and Bcl-XL, inhibit pore openingwhile proapoptotic Bcl-2 family members, such as Bax, Bak, and Bid, can induce MPT pore opening). Also, MPT pore opening can be inhibited by CypD ligands, such as cyclosporin A (CsA). The opening of MPTP leads to a collapse of transmembrane mitochondrial transmembrane potential and favors the release of apoptogenic proteins, such as cytochrome c (Cyt c). **(B)** A large component of DOX induced cardiotoxicity is mediated by a redox cycle on mitochondrial complex I. Increased ROS generation by DOX redox cycle has several negative consequences, such as mitochondrial transmembrane potential disruption, MPTP formation, ATP depletion, and peroxidation of cellular membranes. Marked mitochondrial morphological disturbances induced by DOX include cristae disruption, matrix disorganization, and mitochondrial fragmentation. MPTP -induced outer membrane rupture due to osmotic swelling or permeabilization of the mitochondrial outer membrane mediated by proapoptotic proteins including BAX can lead to the release of cyt c and AIF. DOX also interferes with topoisomerase II, inhibiting DNA replication and preventing the repair of damage DNA strands. Finally, persistent downregulation of gene expression can be another consequence of DOX toxicity. All of these events may lead to cell death. Reproduced with permission from Carvalho et al. (2014), Copyright 2013 Wiley Periodicals, Inc.

FIGURE 5 Anthracycline drugs can cause mitochondrial dysfunction by inhibiting the activity of mitochondrial transcription enzymes. These transcription enzymes mainly include Top1mt and mitochondrial transcription factors, and mitochondrial transcription factors include peroxisome proliferator-activated receptor-gamma coactivator-1α and 1β, Nuclear respiratory factor-1, mitochondrial transcription factor A (TFAM), and Tumor suppressor protein 53. Reproduced with permission from Schirone et al. (2022), licensed under CC BY 4.0.

FIGURE 6 Anthracycline-induced cell apoptosis is mitochondria-mediated intrinsic signaling. When anthracycline drugs enter cardiac myocytes, they cause the release of Cyt c from the mitochondrial inner membrane, which then binds to Apaf-1 to form the apoptosome. The apoptosome activates the caspase family, leading to cell apoptosis. AKT, as a key factor in the apoptosis signaling pathway, can inhibit cell apoptosis by regulating the Bcl-2 family. In addition to the mitochondria-mediated intrinsic signaling pathway, anthracycline drugs can also induce programmed necrosis and cell pyroptosis, resulting in damage to cardiac myocytes. Reproduced with permission from Anjos et al. (2021), Copyright 2021 Elsevier Inc. All rights reserved.

FIGURE 7 Contributors to Anthracycline -induced cardiovascular toxicity. A combination of clinical and genetic risk factors leads to increased risk of developing toxicity upon cancer therapy treatment. Elucidation of genetic contributors of cancer therapy-induced cardiovascular toxicity facilitates understanding of its molecular mechanism and development of its therapeutic strategies. Reproduced with permission from Kim et al. (2022), Copyright 2022 Elsevier Ltd. All rights reserved.

The corresponding **References** appear below and have been added to the reference list.

The original article has been updated.
